# Game Changers: Blockbuster Small-Molecule Drugs Approved by the FDA in 2024

**DOI:** 10.3390/ph18050729

**Published:** 2025-05-15

**Authors:** Zhonglei Wang, Xin Sun, Mingyu Sun, Chao Wang, Liyan Yang

**Affiliations:** 1 Key Laboratory of Green Natural Products and Pharmaceutical Intermediates, Colleges and Universities of Shandong Province, School of Chemistry and Chemical Engineering, Qufu Normal University, Qufu 273165, China; wchaoqfnu@163.com; 2 School of Pharmaceutical Sciences, Key Laboratory of Bioorganic Phosphorus, Chemistry & Chemical Biology (Ministry of Education), Tsinghua University, Beijing 100084, China; 3 School of Physics and Physical Engineering, Qufu Normal University, Qufu 273165, China; sxinqfnu@163.com (X.S.); smyuqfnu@163.com (M.S.); 4 Beijing National Laboratory for Molecular Sciences, Institute of Chemistry, Chinese Academy of Sciences, Beijing 100190, China

**Keywords:** innovative advancements, small molecule drugs, FDA, first-in-class, breakthrough therapies

## Abstract

This article profiles 27 innovative advancements in small-molecule drugs approved by the U.S. Food and Drug Administration (FDA) in 2024. These drugs target various therapeutic areas including non-small cell lung cancer, advanced or metastatic breast cancer, glioma, relapsed or refractory acute leukemia, urinary tract infection, *Staphylococcus aureus* bloodstream infections, nonalcoholic steatohepatitis, primary biliary cholangitis, Duchenne muscular dystrophy, hypertension, anemia due to chronic kidney disease, extravascular hemolysis, primary axillary hyperhidrosis, chronic obstructive pulmonary disease, severe alopecia areata, WHIM syndrome, Niemann–Pick disease type C, schizophrenia, supraventricular tachycardia, congenital adrenal hyperplasia, and cystic fibrosis. Among these approved small-molecule drugs, those with unique mechanisms of action and designated as breakthrough therapies by the FDA represent a significant proportion, highlighting ongoing innovation. Notably, eight of these drugs (including Rezdiffra^®^, Voydeya^®^, Iqirvo^®^, Voranigo^®^, Livdelzi^®^, Miplyffa^®^, Revuforj^®^, and Crenessity^®^) are classified as “first-in-class” and have received breakthrough therapy designation. These agents not only exhibit distinct mechanisms of action but also offer substantial improvements in efficacy for patients compared to prior therapeutic options. This article offers a comprehensive analysis of the mechanisms of action, clinical trials, drug design, and synthetic methodologies related to representative drugs, aiming to provide crucial insights for future pharmaceutical development.

## 1. Introduction

In 2024, the field of drug discovery continues to demonstrate substantial productivity [1,2,3,4,5,6]. The U.S. Food and Drug Administration (FDA) approved 50 new molecular entity (NME) drugs this year, representing a decline from the 55 approvals recorded in 2023; however, it marks an increase compared to the 38 approvals in 2022 [7]. This article reviews the latest research developments on 27 small-molecule drugs (Table 1), including Exblifep^®^ (1), Rezdiffra^®^ (2), Tryvio^®^ (3), Duvyzat^®^ (4), Vafseo^®^ (5), Voydeya^®^ (6), Zevtera^®^ (7), Ojemda^®^ (8), Xolremdi^®^ (9), Iqirvo^®^ (10), Sofdra^®^ (11), Ohtuvayre^®^ (12), Leqselvi^®^ (13), Voranigo^®^ (14), Livdelzi^®^ (15), Lazcluze^®^ (16), Miplyffa^®^ (17), Aqneursa^®^ (18), Cobenfy^®^ (19), Itovebi^®^ (20), Orlynvah^®^ (21), Revuforj^®^ (22), Rapiblyk^®^ (23), Attruby^®^ (24), Crenessity^®^ (25), Ensacove^®^ (26), and Alyftrek^®^ (27). They demonstrate a range of pharmaceutical activities, predominantly as anti-cancer agents (8, 14, 16, 20, 22, 26), genetic drugs (4, 17, 18, 24, 25, 27), immunological drugs (9, 10, 13, 15), and anti-infective drugs (1, 7, 21). Other therapeutic disorders include endocrine/metabolic drugs (2, 11), cardiovascular/cerebrovascular drugs (3, 23), hematological drugs (5, 6), respiratory drugs (12), and psychotropic drugs (19).

Among the approved small-molecule drugs, those distinguished by unique mechanisms of action and those designated as breakthrough therapies by the FDA constitute a significant proportion, highlighting ongoing innovation in this field [8]. Notably, eight of these drugs—Rezdiffra, Voydeya, Iqirvo, Voranigo, Livdelzi, Miplyffa, Revuforj, and Crenessity—are classified as “first-in-class” and have received breakthrough therapy designation. These agents not only demonstrate distinct mechanisms of action but also provide substantial improvements in efficacy for patients compared to previous therapeutic options. This article presents a chronological and comprehensive analysis of the drug design processes, synthetic methodologies, mechanisms of action, and clinical trials associated with representative pharmaceuticals. This article aims to provide critical insights that will inform future drug development endeavors.

## 2. Exblifep (Cefepime/Enmetazobactam)

**Scheme 1:**
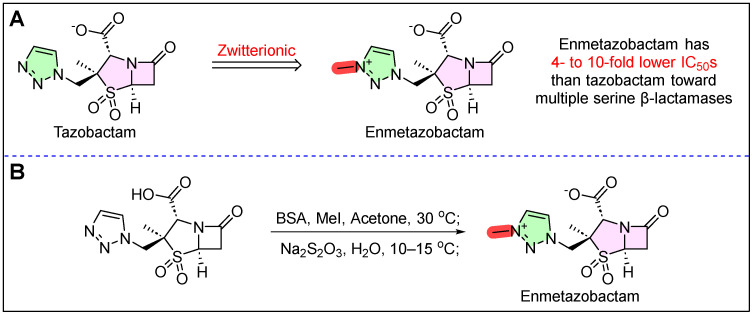
The discovery (**A**) and synthesis (**B**) of enmetazobactam.

On 22 February 2024, the FDA approved Exblifep, an intravenous (IV) antibacterial combination therapy developed by Allecra that integrates cefepime and enmetazobactam [9]. This treatment is for complicated urinary tract infections (cUTIs) in patients aged 18 years and older, including those with pyelonephritis. Cefepime, a *β*-lactam antibiotic initially developed by Bristol-Myers Squibb and approved in 1994, was removed from the WHO’s essential medicines list in 2019 due to decreased efficacy against resistant pathogens [10]. In drug research, replacing hydrogen atoms with methyl groups is a common strategy to improve small-molecule properties [11,12] (Scheme 1). The introduction of a methyl group to tazobactam results in the formation of a zwitterionic variant, enmetazobactam, which demonstrates IC_50_ values that are 4- to 10-fold lower than those observed for tazobactam against various serine *β*-lactamases [11,12] (Scheme 1). Enmetazobactam functions as a *β*-lactamase inhibitor that safeguards cefepime from degradation by specific serine *β*-lactamases such as extended-spectrum *β*-lactamases [13]. These inhibitors are specifically designed to counter resistance mechanisms employed by pathogens to evade cefepime and other *β*-lactams. Enmetazobactam has demonstrated effectiveness against “superbugs,” including Enterobacteriaceae and carbapenem-resistant *Pseudomonas aeruginosa* [14,15]. Merck employs a similar strategy with Recarbrio (imipenem/cilastatin-relebactam), which received FDA approval in 2019 for treating cUTIs and intra-abdominal infections [16].

The approval of Exblifep is based on a double-blind, non-inferiority Phase III trial (NCT03687255) involving 1041 adults diagnosed with complicated urinary tract infections, including pyelonephritis [17]. Participants were randomly assigned in a 1:1 ratio to receive either cefepime (2 g)/enmetazobactam (500 mg) (*n* = 345) or piperacillin (4 g)/tazobactam (500 mg) (*n* = 333), administered every eight hours for up to fourteen days for those presenting with concurrent bacteremia. The primary efficacy endpoint was defined as a composite response encompassing both clinical cure and microbiological eradication at the test-of-cure visit. Results indicated that the composite response rate was significantly higher in the cefepime/enmetazobactam group, achieving a rate of 79.1% (273/345), compared to a rate of 58.9% (196/333) in the piperacillin/tazobactam group, yielding an adjusted stratified difference of 21.2% [95% CI, 14.3–27.9]. Among patients exhibiting baseline bacteremia, the response rate also favored the cefepime/enmetazobactam group at 71% (27/38), versus only 50% (14/28) in the comparator group. For subjects harboring extended-spectrum β-lactamase-producing bacteria at baseline, responses were observed in 74% (56/76) of those treated with cefepime/enmetazobactam compared to just 52% (34/66) among those receiving piperacillin/tazobactam. Common adverse reactions associated with cefepime/enmetazobactam included elevated transaminases, increased bilirubin levels, headache, and venous inflammation or infusion site reactions.

## 3. Rezdiffra (Resmetirom)

Non-alcoholic steatohepatitis (NASH), recently redefined as metabolic dysfunction-associated fatty liver disease (MASH), is a rare hepatic condition that frequently coexists with metabolic disorders such as diabetes and obesity [18,19]. This condition is characterized by the accumulation of fat in the liver, which leads to inflammation and fibrosis. NASH/MASH is often called a “silent disease” due to its asymptomatic nature; however, it can progress over time as fat accumulation induces inflammation and fibrosis, ultimately advancing through four stages that culminate in cirrhosis. The precise prevalence of MASH remains uncertain because it typically presents without symptoms. On March 14, Madrigal Pharmaceuticals announced that the U.S. FDA granted accelerated approval for Rezdiffra (resmetirom [MGL-3196]), marking it as the first treatment specifically indicated for adults with non-cirrhotic NASH who exhibit moderate to severe liver fibrosis (F2 to F3) [20]. Rezdiffra is currently the only FDA-approved medication for the treatment of MASH [21]. This oral thyroid hormone receptor beta (THR-β) agonist is administered once daily and selectively targets THR-β in the liver, addressing the underlying causes of MASH by mediating metabolic processes such as reducing lipid levels while avoiding the activation of THR-α, thereby alleviating associated safety concerns.

**Scheme 2:**
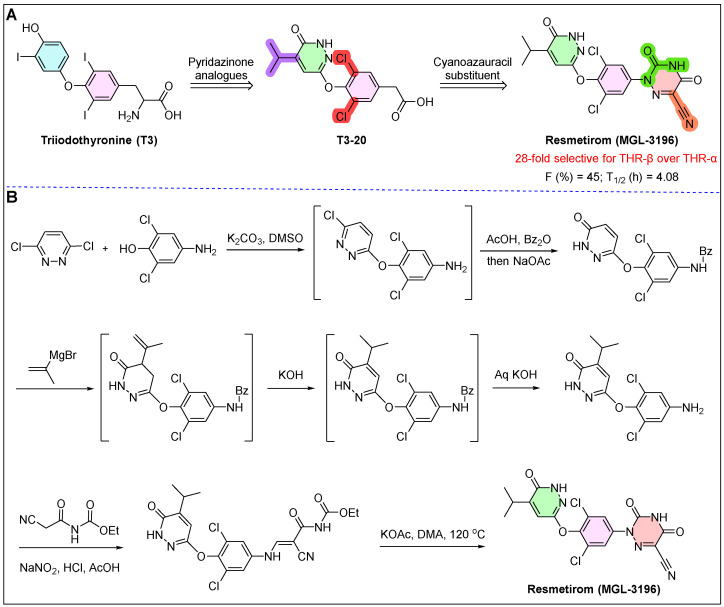
The discovery (**A**) and synthesis (**B**) of resmetirom.

The beneficial metabolic effects of triiodothyronine (T3) on cholesterol and triglyceride levels have been recognized for many years [22]. However, the interaction of thyroid hormones with the thyroid hormone receptor alpha subtype (THR-α) can result in adverse effects on cardiac function and bone health [23]. To optimize this series, a cyanourea substituent was introduced, which enhanced both the titer and selectivity—specifically, the selectivity of THR-β is 28 times greater than that of THR-α—ultimately leading to the development of resmetirom [24,25,26] (Scheme 2).

The approval of Rezdiffra is based on a 12-month efficacy analysis derived from the ongoing 54-month randomized, double-blind, placebo-controlled Phase III trial known as MAESTRO-NASH (NCT03900429) [27]. This study represents one of four Phase III trials within the clinical development program for resmetirom, with results up to 12 months. A total of 888 patients exhibiting initial fibrosis stages F2 or F3 were enrolled and randomly assigned in a ratio of 1:1:1 to receive either Rezdiffra at a dosage of 80 mg once daily (*n* = 298), Rezdiffra at a dosage of 100 mg once daily (*n* = 296), or placebo (*n* = 294). Efficacy was evaluated at the end of the 12-month period through post-treatment liver biopsy outcomes, specifically focusing on the resolution of steatohepatitis without the exacerbation of fibrosis and one-stage improvement in fibrosis without deterioration in steatohepatitis. A key secondary endpoint involved assessing the percentage change in low-density lipoprotein cholesterol from baseline at week 24. Both dosages of Rezdiffra demonstrated significant improvements in these histopathological endpoints when compared to placebo. Statistical analyses that incorporated independent evaluations by two pathologists confirmed significance for both dosages across both endpoints. From month three to month twelve, participants receiving Rezdiffra exhibited greater reductions in average ALT and AST levels than those receiving placebo; elevated ALT and AST levels are indicative of liver impairment. Regarding secondary endpoints, changes observed in low-density lipoprotein cholesterol levels by week twenty-four were −13.6% for the Rezdiffra 80 mg group, −16.3% for the Rezdiffra 100 mg group, and +0.1% for the placebo group. In terms of safety, the most frequently reported adverse reactions among patients treated with Rezdiffra include diarrhea, nausea, itching, abdominal pain, vomiting, constipation, and dizziness. Diarrhea and nausea typically manifest early in the treatment process and can range from mild to moderate severity. Patients with decompensated cirrhosis are advised to avoid Rezdiffra. The FDA’s approval encompasses three dosage levels based on patient weight: 80 mg for individuals weighing less than 100 kg (220 pounds) and 100 mg for those exceeding this weight threshold. Given that this was an accelerated approval by the FDA, additional data from confirmatory studies are required.

## 4. Tryvio (Aprocitentan)

Hypertension is characterized by elevated vascular pressure, defined as 140/90 mmHg or higher, and its prevalence continues to rise [28,29,30]. The number of individuals affected by hypertension has nearly doubled over the past four decades, with approximately 1.13 billion people worldwide suffering from this condition [31]. The World Health Organization estimates that hypertension is responsible for around 7.5 million deaths annually, accounting for about 12.8% of global mortality rates [31]. Typically, hypertension develops insidiously over many years without noticeable symptoms; if left untreated, it can lead to severe complications such as stroke, blindness, and heart failure [32]. Despite advancements in antihypertensive therapies in recent years, the last approved drug featuring a new mechanism was more than three decades ago [33,34]. On 19 March 2024, the U.S. FDA granted approval for Tryvio (active ingredient: Aprocitentan), an NME developed by Idorsia Pharmaceuticals [35]. Tryvio functions as an endothelin receptor antagonist and represents the first orally administered antihypertensive therapy approved through a new treatment pathway in nearly thirty years [36]. Prior to Tryvio’s approval, there were no FDA-approved antihypertensive therapies specifically targeting the endothelin (ET) pathway. Aprocitentan inhibits the binding of endothelin-1 (ET-1) to both endothelin receptor A (ETA) and B (ETB) [37]. ET-1 is a peptide composed of 21 amino acids recognized as the most potent and long-lasting vasoconstrictor currently identified; it also contributes to endothelial dysfunction, vascular hypertrophy and remodeling, sympathetic nervous system activation, and increased aldosterone synthesis [38].

**Scheme 3:**
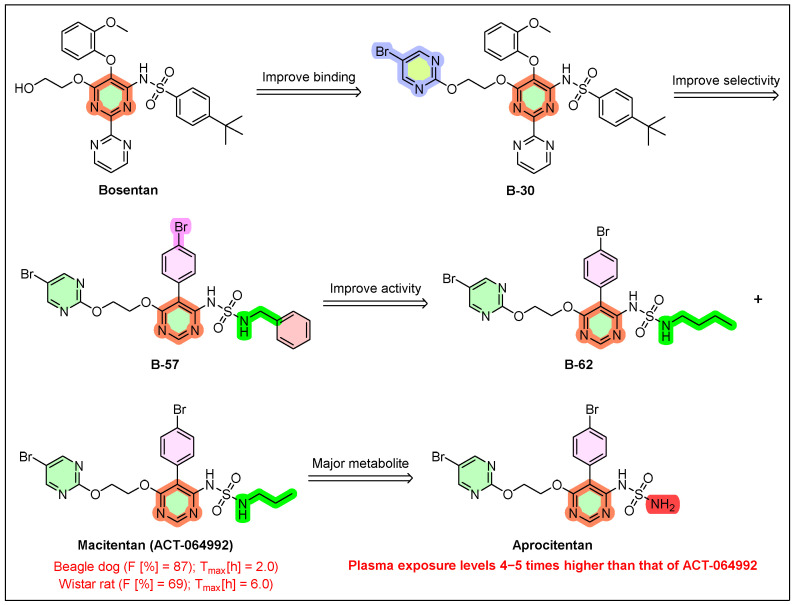
The discovery of aprocitentan, a major metabolite of ACT-064992.

**Scheme 4:**
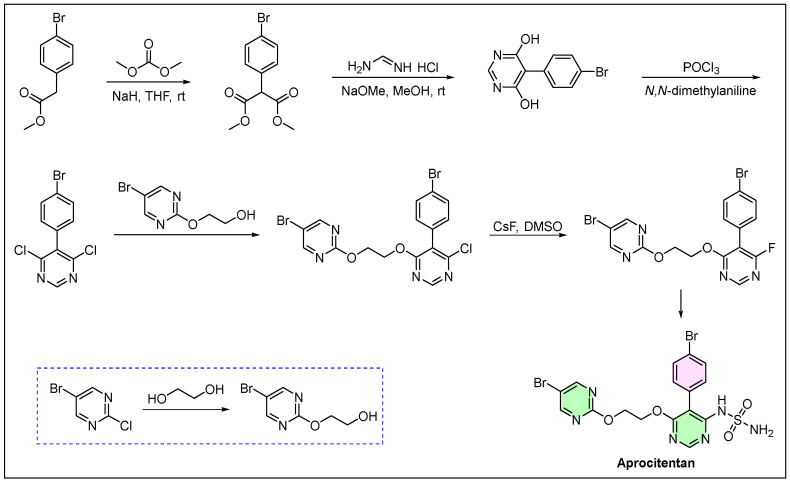
Synthesis of aprocitentan.

Beginning with bosentan, Bolli et al. [39] launched a medicinal chemistry program to identify novel and potent dual endothelin receptor antagonists with high oral bioavailability. This effort led to the discovery of a new series of alkyl sulfamide-substituted pyrimidines. Notably, macitentan (ACT-064992) emerged as an effective ETA inhibitor, exhibiting strong affinity for the ETB receptor and excellent pharmacokinetic properties, leading to the discovery of aprocitentan [39] (Scheme 3 and Scheme 4). The FDA’s approval of Tryvio was based on the Phase III PRECISION trial, which assessed both its short-term and long-term efficacy in a cohort of 730 patients with difficult-to-control hypertension across three distinct phases [40]. The initial phase comprised a 4-week double-blind period during which participants were randomly assigned to receive either 12.5 mg (*n* = 243) or 25 mg (n = 243) of Aprocitentan or a placebo (*n* = 244). The second phase involved a single-blind period lasting 32 weeks (weeks 4–36), wherein patients received 25 mg of Aprocitentan (*n* = 704). The third phase entailed a 12-week double-blind withdrawal period, during which patients were re-randomized to receive either 25 mg of Aprocitentan (*n* = 307) or a placebo (*n* = 307). Results demonstrated that Tryvio achieved the primary efficacy endpoint by significantly reducing seated systolic blood pressure (SBP) compared to the placebo group after four weeks. Furthermore, it met key secondary endpoints as SBP remained lower in the Aprocitentan group during weeks 36–40, while the SBP in the placebo group increased by +5.8 mmHg at week 40. The approval of Tryvio followed years of rigorous preclinical and clinical research, with favorable outcomes from the Phase III trial (NCT03541174) published in *The Lancet* facilitating its market introduction [41]. Idorsia submitted the new drug application for Tryvio to the FDA on 19 December 2022; subsequent review culminated in its approval for market release on 19 March 2024 [35]. This approval addresses an existing gap in systemic antihypertensive therapies targeting the endothelin pathway and offers a novel treatment option for patients suffering from hypertension.

## 5. Duvyzat (Givinostat)

**Scheme 5:**
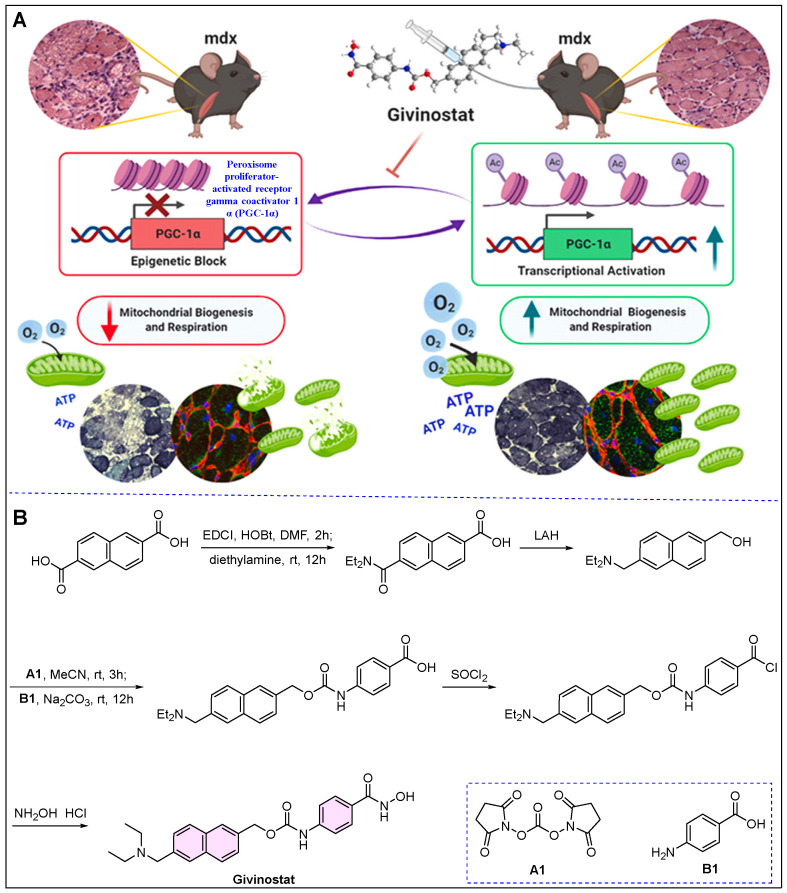
(**A**). Adapted from Giovarelli et al., 2021 [46]. Copyright © 2021, with permission from Elsevier. (**B**) Synthesis of givinostat, which enhances mitochondrial biogenesis in DMD.

Duchenne muscular dystrophy (DMD) is a rare neuromuscular disorder characterized by progressive muscle weakness resulting from dystrophin deficiency [42]. Mothers of affected children are typically carriers of the pathogenic gene, which confers a 50% risk to male infants of developing the disease. DMD affects approximately 1 in every 3600 male newborns globally. As the condition progresses, patients gradually lose their ability to walk and experience diminishing muscle strength, potentially leading to respiratory complications and premature mortality [43]. On 21 March 2024, the U.S. FDA granted approval for Duvyzat (givinostat), developed by Italfarmaco S.p.A. in Milan, Italy, as a treatment option for patients aged six years and older with DMD [44,45]. Regarding its mechanism, Palm et al. [46] showed that givinostat significantly influences mitochondria, acting as a metabolic remodeling agent that enhances mitochondrial biogenesis in malnourished muscle. Duvyzat acts as a histone deacetylase (HDAC) inhibitor; while its exact mechanism of action in treating DMD is still unclear, studies have revealed increased HDAC activity in these patients. By inhibiting this enzyme, the drug aims to mitigate excessive pathological activity associated with it. This approval represents the first instance of a non-steroidal medication being sanctioned by the FDA for all individuals possessing mutations in the DMD gene [46,47] (Scheme 5).

The approval of Duvyzat is predicated on clinical data derived from the Phase III EPIDYS trial (NCT02851797), which successfully achieved its primary and key secondary endpoints [48]. Throughout the 18-month treatment period, patients who were administered Duvyzat demonstrated a significant reduction in the time required to ascend four stairs, with a decrease from baseline to 1.25 s at 18 months, in contrast to 3.03 s observed in placebo recipients. Furthermore, the Duvyzat cohort exhibited substantial improvements across secondary measures, including North Star Ambulatory Assessment (NSAA) scores, time to stand, muscle strength evaluations, and magnetic resonance imaging assessments of fat infiltration. Most adverse reactions reported were classified as mild to moderate; commonly noted side effects included diarrhea, abdominal pain, thrombocytopenia, nausea/vomiting, hypertriglyceridemia, and fever.

## 6. Vafseo (Vadadustat)

**Scheme 6:**
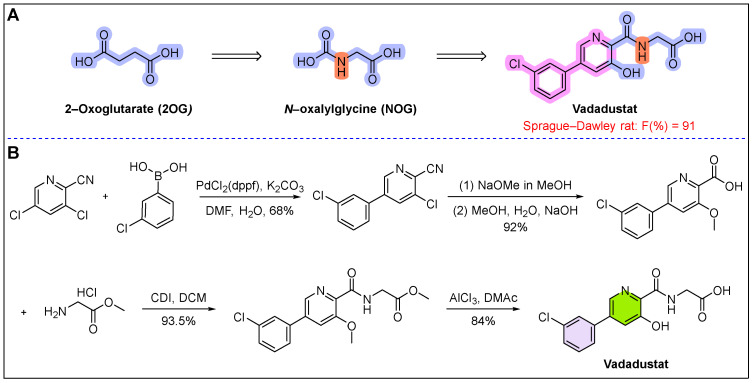
The discovery (**A**) and synthesis (**B**) of vadadustat.

Chronic kidney disease (CKD) represents a significant global health challenge, with a 29.3% increase in prevalence over the past three decades, currently affecting more than 697.5 million individuals worldwide [49,50]. Anemia is a prevalent complication among patients with CKD, with its incidence escalating as renal function deteriorates [51]. On 27 March 2024, the U.S. FDA granted approval for Vafseo (active ingredient: Vadadustat), developed by Akebia Therapeutics. Vafseo is classified as a hypoxia-inducible factor prolyl hydroxylase (HIF-PHD) inhibitor and is indicated for the treatment of anemia associated with CKD in adults who have been on dialysis for at least three months [8]. This medication activates the physiological response to hypoxia by stimulating endogenous erythropoietin production and effectively managing anemia. Vafseo was initially approved in Japan on 29 June 2020 and has since received authorization in 37 countries/regions following FDA approval [52]. As an HIF-PHD inhibitor featuring a pyridine core structure, it stabilizes HIF under low oxygen conditions through the inhibition of prolyl hydroxylase (PHD) [53]. This mechanism results in elevated levels of endogenous erythropoietin and therapeutic benefits. HIF-PH activity is influenced by iron and 2-oxoglutaric acid (2OG). Following the discovery of N-oxalate glycine (NOG), subsequent investigations into vadadustat have identified NOG as the primary active binding site [54] (Scheme 6). Vadadustat has high oral bioavailability, estimated at 91% for a 20 mg/kg dose across sexes in Sprague–Dawley rats [55,56].

The FDA’s approval was based on the results of two Phase III clinical trials, INNO2VATE-1 (NCT02865850) and INNO2VATE-2 (NCT02892149), designed as randomized, active-controlled, non-inferiority studies [57]. Both trials evaluated the efficacy and safety of Vadadustat in treating anemia among patients with dialysis-dependent chronic kidney disease (DD-CKD). A total of 3923 DD-CKD patients aged between 19 and 93 years participated; among them, 55.9% were male. The ethnic distribution included 64.5% Caucasian, 38.5% Hispanic, 24.1% Black (including African American), and 4.5% Asian. Patients were randomly assigned in a ratio of 1:1 to receive either an initial daily dose of Vafseo at 300 mg or Darbepoetin Alfa (an erythropoietin receptor agonist used as a control). The administration was conducted subcutaneously or intravenously over a period of 52 weeks to evaluate the efficacy endpoints associated with Vafseo. Following this treatment duration, participants continued receiving therapy to assess long-term safety until major adverse cardiovascular events (MACEs) occurred. Results demonstrated that Vafseo was not inferior to Darbepoetin Alfa in correcting and maintaining hemoglobin levels during weeks 24 to 36 and weeks 40 to 52 across both trials.

Although Vafseo has been launched in many countries, its approval journey in the United States has encountered significant challenges. Akebia submitted a New Drug Application (NDA) for vadadustat to the FDA on 28 March 2021; however, this application was rejected on 29 March 2022. The FDA concluded that the risks associated with vadadustat outweighed its benefits due to insufficient evidence demonstrating non-inferiority in reducing major adverse cardiovascular event (MACE) risk among non-dialysis patients and an increased incidence of thromboembolic events as well as drug-induced liver injury in dialysis patients. On 27 September 2023, Akebia resubmitted a New Drug Application for vadadustat, incorporating data from two pivotal Phase III clinical trials and addressing the concerns raised during the previous review. Following a comprehensive evaluation, the FDA granted marketing approval for vadadustat in the U.S. while also issuing a boxed warning regarding elevated risks of death, myocardial infarction, stroke, venous thromboembolism, and thrombosis at vascular access sites. In summary, with FDA approval now secured, Vafseo is authorized for use in 37 countries. Its mechanism of action differs from traditional anemia treatments such as iron supplements and erythropoiesis-stimulating agents; its therapeutic efficacy is comparable to that of Darbepoetin Alfa used clinically. Ongoing clinical studies involving Vafseo are anticipated to yield further insights into its safety and effectiveness.

## 7. Voydeya (Danicopan)

**Scheme 7:**
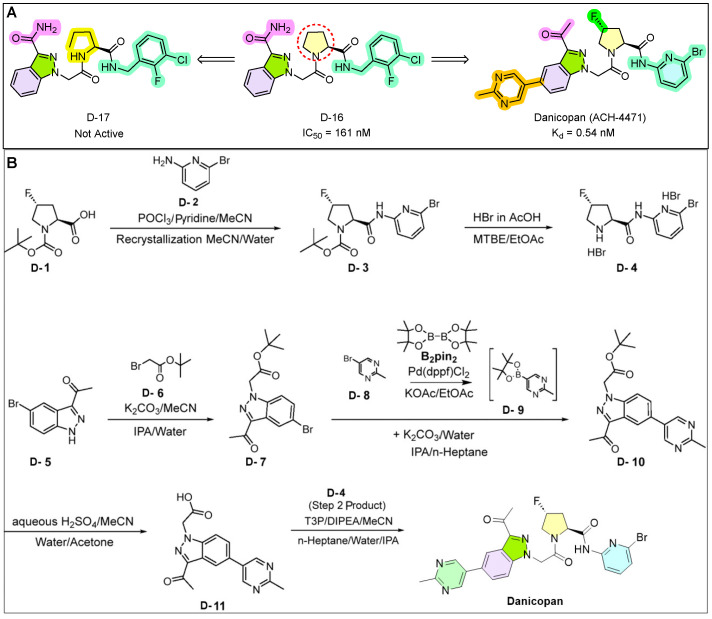
The discovery (**A**) and synthesis (**B**) of danicopan.

Paroxysmal nocturnal hemoglobinuria (PNH) is a rare, chronic, and potentially life-threatening hematological disorder caused by genetic mutations that lead to the absence of complement regulatory proteins CD55 and CD59 on red blood cells [58]. This deficiency activates the alternative pathway of the complement cascade, resulting in intravascular hemolysis and subsequent damage to red blood cells. Furthermore, this activation can also stimulate leukocytes and platelets, thereby increasing the risk of thrombosis. Currently, C5 complement inhibitors represent the primary treatment modality for PNH; however, many patients continue to experience anemia and may require blood transfusions [59]. On March 29, AstraZeneca announced that its oral complement factor D inhibitor, Voydeya (danicopan), has received approval from the U.S. FDA as an adjunct therapy for adults with PNH who are experiencing extravascular hemolysis (EVH), intended for use alongside C5-targeted monoclonal antibodies [60]. Voydeya is classified as a small-molecule drug that complements standard therapies such as Ultomiris (ravulizumab) or Soliris (eculizumab) in treating EVH among adult PNH patients [61]. It functions by selectively inhibiting complement factor D within the alternative pathway, thereby reducing premature red blood cell breakdown through the blockade of C3 convertase production and the suppression of alternative pathway activity. Unlike C5 inhibitors, Voydeya effectively prevents the deposition of C3b fragments on red blood cells in patients with paroxysmal nocturnal hemoglobinuria (PNH). This mechanism plays a crucial role in controlling red blood cell breakdown and extravascular hemolysis (EVH) during treatment with C5 inhibitors. Through high-throughput screening, researchers identified L-proline-based compounds that inhibit complement factor D. Modifications to the amide substituents of L-proline led to D-16, where the proline ring-opening causes inactivation (D-17). Ultimately, danicopan was developed using structure-based drug design and fragment-based drug design to optimize substituent selection at each site [62,63,64] (Scheme 7).

The approval of Voydeya is predicated on the favorable outcomes from the randomized, placebo-controlled Phase III study ALPHA [60]. This investigation encompassed 84 patients exhibiting clinically significant extravascular hemolysis attributable to paroxysmal nocturnal hemoglobinuria. The results demonstrated that participants in the danicopan group experienced statistically and clinically significant enhancements in hemoglobin levels at 12 weeks when compared to the placebo cohort, thereby fulfilling both primary and secondary endpoints. Data indicated that Voydeya was generally well tolerated, with no novel safety concerns identified. There were no grade 4 or 5 treatment-emergent adverse events (TEAEs). The most frequently reported TEAEs within the treatment group included headache (10.2%), nausea (8.2%), joint pain (8.2%), and diarrhea (8.2%), underscoring a favorable safety profile.

## 8. Zevtera (Ceftobiprole)

**Scheme 8:**
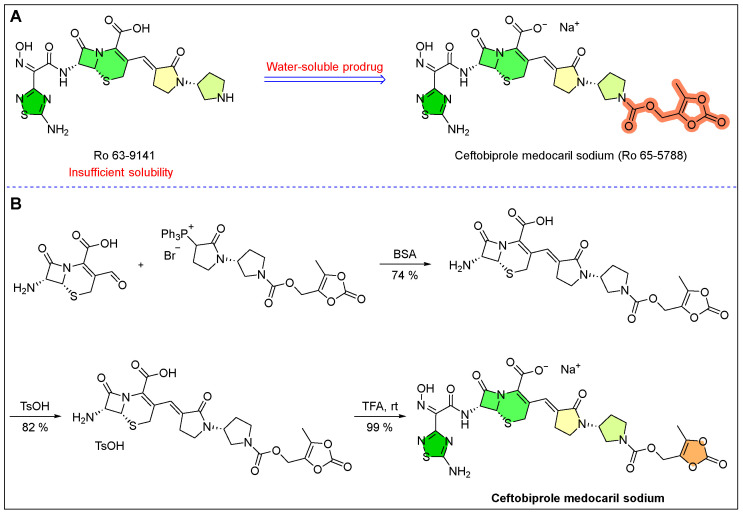
The discovery (**A**) and synthesis (**B**) of ceftobiprole.

In the past 15 years, Zevtera has received approval in over 80 countries, including China, Canada, and most European nations, for the treatment of various infections [2]. On 3 April 2024, the U.S. FDA granted approval for Zevtera (ceftobiprole medocaril sodium) as an injectable formulation to treat *Staphylococcus aureus* bacteremia (SAB), acute bacterial skin and skin structure infections (ABSSSIs), and community-acquired bacterial pneumonia (CABP) [65]. This represents the first β-lactam antibiotic approved by the FDA for SAB in a span of 15 years, thereby introducing a novel therapeutic option for these severe infectious diseases. The approval was based on data derived from three Phase III clinical trials: ERADICATE (NCT03138733) [66], TARGET (NCT03137173) [67], and a CABP study (NCT00326287) [68]. Ro 63-9141, a novel parenteral cephalosporin with broad-spectrum activity against Gram-positive and Gram-negative pathogens, is the active principle of water-soluble prodrug ceftobiprole (Ro 65-5788) [69,70,71] (Scheme 8).

SAB refers to the presence of *Staphylococcus aureus* bacteria in the bloodstream, which can occur spontaneously through pathways such as the urinary tract or intravenous catheterization. Bacteremia has the potential to lead to metastatic infections, including endocarditis. Cryptic bacteremia often presents with no overt symptoms but can manifest as fever. A randomized, double-blind, multicenter Phase III clinical trial (NCT03138733) was conducted to evaluate the efficacy and safety of Zevtera compared to Daptomycin for treating *Staphylococcus aureus* bacteremia (including infectious endocarditis) [66]. This study was initiated by Basilea on 26 August 2018 and completed on 11 March 2022; it enrolled a total of 390 patients diagnosed with SAB. Participants were randomly assigned to receive either Zevtera (192 subjects) or Daptomycin (198 subjects). The Zevtera group received intravenous injections every six hours for the first eight days, followed by every eight hours thereafter; conversely, participants in the Daptomycin group received injections once every twenty-four hours. Clinical outcomes indicated that overall treatment success was achieved in 69.8% of patients receiving Zevtera compared to 68.7% in those receiving Daptomycin. Safety profiles revealed similar rates of adverse events between both groups; however, gastrointestinal adverse events were reported slightly more frequently among patients treated with Zevtera. These findings suggest that the therapeutic effect of Zevtera is not inferior to that of Daptomycin.

ABSSSI represents significant bacterial infections of the skin. Over the past two decades, there has been a global increase in diagnosed cases. Common complications associated with these infections include secondary joint infections, bloodstream infections (BSIs), and amputations; among these, BSI is particularly concerning due to its potential to result in multiple organ dysfunction or septic shock. A randomized, double-blind Phase III clinical trial (NCT03137173) was conducted to compare the efficacy and safety of Zevtera versus Vancomycin for the treatment of ABSSSI. This study took place from 19 February 2018 to 22 April 2019, enrolling a total of 679 patients—335 received Zevtera while 344 were treated with Vancomycin plus Aztreonam [67]. The early clinical success rate was found to be 91.3% in the Zevtera group compared to 88.1% in the control group (adjusted difference: 3.3%; 95% CI: −1.2 to 7.8), thereby confirming non-inferiority. Success rates at test of cure (TOC) were comparable between groups: within the Intent-to-Treat population, success rates were recorded at 90.1% for Zevtera versus 89.0% for controls; similarly, within the clinically evaluable population, rates stood at 97.9% for Zevtera against 95.2% for controls. Both treatments exhibited similar success rates and safety profiles throughout this investigation. These findings indicate that the therapeutic effect of Zevtera is not inferior to that provided by Vancomycin/Aztreonam.

CABP represents a significant health concern, with approximately one in five patients necessitating hospitalization and an associated mortality rate of 8%. A prompt initiation of antibiotic therapy targeting potential pathogens is critical within hours following diagnosis, as delays or the use of ineffective antibiotics can exacerbate morbidity and mortality. A randomized, double-blind, multicenter Phase III clinical trial (NCT00326287) was conducted to evaluate the efficacy of Zevtera compared to placebo for the treatment of CABP [68]. This study took place from June 2006 to July 2007 and enrolled a total of 638 participants: 314 were administered Zevtera, while 324 received Ceftriaxone. The results indicated that clinical cure was achieved in 76.4% of patients receiving Zevtera, compared to 79.3% among those treated with the control drug. Notably, an earlier assessment at day three revealed comparable outcomes between both groups: a clinical cure rate of 71% for Zevtera versus 71.1% for Ceftriaxone.

In summary, the approval of Zevtera signifies a substantial advancement within the medical community. As a novel generation cephalosporin antibiotic, it exhibits rapid bactericidal activity and demonstrates efficacy against a range of Gram-positive bacteria, including methicillin-resistant *Staphylococcus aureus*, as well as various Gram-negative bacteria. Nevertheless, further research and clinical practice are essential to comprehensively assess its safety and long-term efficacy for rational application in clinical settings. Overall, the approval of Zevtera represents an important milestone in medicine, providing broader opportunities for future antibacterial therapies.

## 9. Ojemda (Tovorafenib)

**Scheme 9:**
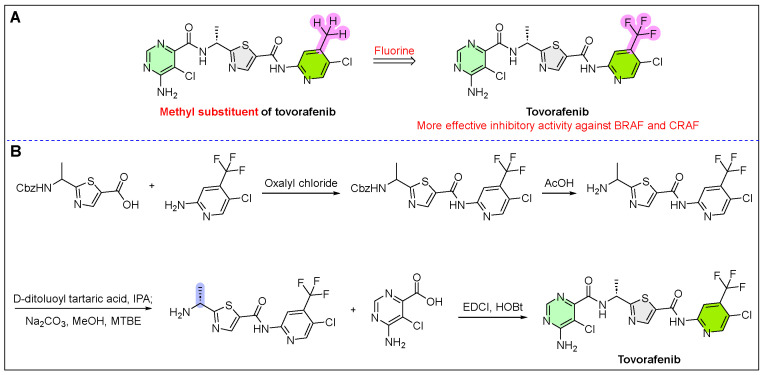
The discovery (**A**) and synthesis (**B**) of tovorafenib.

Pediatric low-grade glioma (pLGG) is the most prevalent central nervous system tumor in children, accounting for approximately 30% of pediatric brain tumors [71]. The ten-year PFS is estimated to be around 50%. pLGG can adversely affect various aspects of children’s health, including vision and motor function. Most genetic mutations associated with pLGG are linked to hereditary alterations in the RAS/mitogen-activated protein kinase (RAS/MAPK) pathway. BRAF fusions occur in 30–40% of these tumors and in 70–80% of pilocytic astrocytomas, with up to 75% of pLGG patients exhibiting BRAF alterations. Currently approved BRAF inhibitors demonstrate limited efficacy against brain tumors and are ineffective for those with BRAF fusions. On 23 April 2024, the U.S. FDA granted accelerated approval for Ojemda (tovorafenib), a pan-RAF kinase inhibitor developed by Day One Biopharmaceuticals, intended for the treatment of recurrent or refractory pLGG in patients aged six months and older [72]. Tovorafenib selectively inhibits tumor growth associated with both BRAF fusions and V600 mutations while demonstrating favorable brain penetrability. Additionally, it has received breakthrough therapy designation as well as orphan drug status from the FDA for treating pLGG characterized by activating RAF mutations. Tovorafenib shows greater inhibitory activity against RAF kinases, including BRAF and CRAF, compared to analogs with a methyl substituent [73,74,75] (Scheme 9).

The FDA’s approval of tovorafenib was predicated on favorable outcomes from the FIREFLY-1 trial, a multinational, multicenter, single-arm Phase II study (NCT04775485) [76]. This open-label trial enrolled 137 patients with recurrent or refractory BRAF-altered pLGG who had previously received at least one systemic treatment. The primary efficacy endpoint was the overall response rate (ORR), defined according to RAPNO-LGG criteria and assessed by blind independent central review (BICR). The ORR was determined as the proportion of patients achieving complete response (CR), partial response (PR), or minimal response (MR). Additional endpoints included the duration of response (DoR). Results indicated an ORR of 67% among evaluable patients, with a CR rate of 17% and a PR rate of 49%, culminating in a clinical benefit rate of 93%. Most patients treated with Ojemda exhibited significant tumor shrinkage or stability. The median DoR was recorded at 16.6 months, suggesting that over half maintained tumor control for this duration following treatment. In patients harboring BRAF fusions, the ORR reached 69%, whereas it stood at 50% for those possessing BRAF V600E mutations; these results underscore Ojemda’s effectiveness against various BRAF mutations in pLGG. Among individuals previously treated with MAPK inhibitors (MAPKi), the ORR attained 71%, compared to a figure of 61% in untreated subjects, indicating sustained efficacy following prior MAPKi therapy. These findings substantiate that Ojemda demonstrates considerable efficacy in managing recurrent or refractory pLGG cases characterized by BRAF mutations. Although some efficacy was observed during treatment, the long-term use of this therapy presents challenges related to safety and the development of resistance. Common adverse reactions include rash, changes in hair color, fatigue, viral infections, vomiting, headache, fever, dry skin, constipation, nausea, acne, and upper respiratory infections.

Ojemda has demonstrated significant rapid and sustained tumor responses in patients with recurrent or refractory pLGG harboring BRAF mutations. Its approval marks a crucial advancement in the medical community’s efforts to treat pediatric brain tumors. This development offers renewed hope for patients with rare pediatric tumors and provides an effective new treatment option for children with pLGG who are in urgent need of novel therapies.

## 10. Xolremdi (Mavorixafor)

**Scheme 10:**
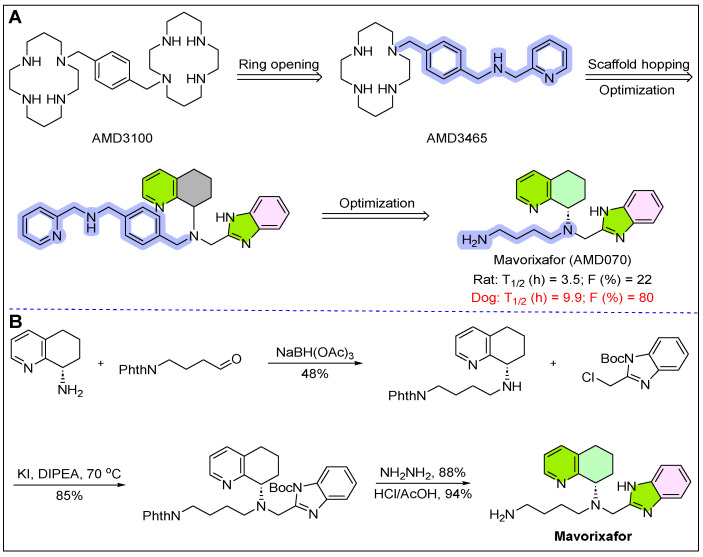
The discovery (**A**) and synthesis (**B**) of mavorixafor.

CXCR4 (also known as CD184) is a chemokine receptor and a seven-transmembrane protein belonging to the G protein-coupled receptor (GPCR) family. It primarily interacts with CXCL12, playing critical roles in cell migration and localization [77]. Dysregulation of the CXCR4 signaling pathway can contribute to cancer growth, invasion, metastasis, and angiogenesis, underscoring its significance in cancer progression, hematopoietic stem cell transplantation, and immune-related diseases. Consequently, targeting CXCR4 represents a promising therapeutic strategy. WHIM syndrome is a rare primary immunodeficiency disorder associated with chronic neutropenia resulting from mutations in the CXCR4 gene that impair receptor function. This dysfunction disrupts leukocyte mobilization from bone marrow to peripheral circulation, leading to various immune-related health complications. On April 29, the U.S. FDA approved X4 Pharmaceuticals’ Xolremdi (Mavorixafor) capsules as the first drug specifically indicated for the treatment of WHIM syndrome [78]. As an oral CXCR4 antagonist that has received breakthrough therapy designation and orphan drug status from both the FDA in 2018 and the European Union in 2019, Mavorixafor signifies a substantial advancement in this field. Mavorixafor was initially developed as an inhibitor of HIV-1. Optimization efforts focused on enhancing the linkers, leading to a more active AMD3465. To further improve its bioactivity and pharmacokinetics, mavaloxifol was synthesized [79,80,81] (Scheme 10).

The FDA’s approval was based on the Phase III clinical trial 4WHIM (ClinicalTrials.gov identifier: NCT03995108), a one-year global, randomized, double-blind, placebo-controlled study involving 31 patients aged 12 and older with WHIM syndrome [82]. Participants were administered either once-daily oral mavorixafor (*n* = 14) or placebo (*n* = 17). Efficacy was measured by improvements in absolute neutrophil count (ANC), absolute lymphocyte count (ALC), and reduction in infections. The trial showed that Xolremdi significantly increased ANC levels above ≥500 cells/μL and ALC levels above ≥1000 cells/μL compared to placebo, with *p*-values < 0.0001. Additionally, the total infection score in the Xolremdi group decreased by about 40% relative to placebo, while the annualized infection rate dropped by 60%. Common adverse reactions associated with mavorixafor include thrombocytopenia, rash, dermatitis, rhinitis, epistaxis, vomiting, and dizziness.

## 11. Iqirvo (Elafibranor)

**Scheme 11:**
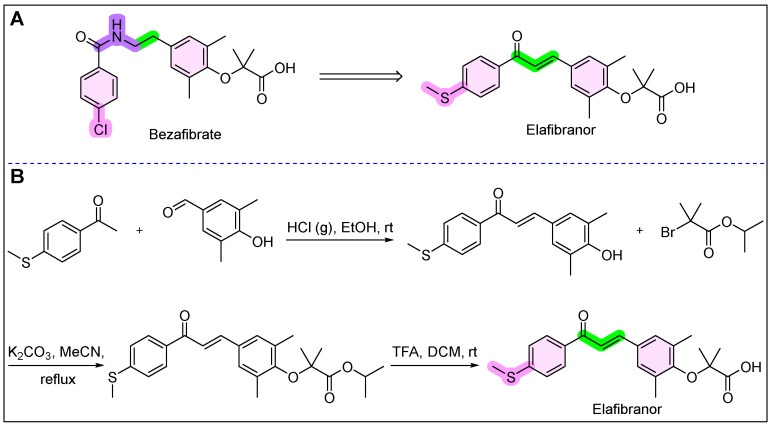
The discovery (**A**) and synthesis (**B**) of elafibranor.

Primary biliary cholangitis (PBC) is a serious autoimmune liver disease characterized by the immune system’s attack on bile ducts, resulting in impaired bile flow and toxic accumulation within the liver [83,84]. This condition can progress to fibrosis, cirrhosis, and ultimately liver failure. Common symptoms include fatigue and pruritus, which are major contributors to the need for liver transplantation. PBC predominantly affects middle-aged women, unfortunately, and many patients do not experience adequate benefits from existing treatments. On June 10, Ipsen announced that the U.S. FDA granted accelerated approval for Iqirvo (elafibranor) 80 mg tablets in combination with ursodeoxycholic acid (UDCA) for adult PBC patients who do not respond sufficiently to UDCA or as monotherapy for those who are intolerant to it [85]. Iqirvo represents a first-in-class oral peroxisome proliferator-activated receptor (PPAR) agonist administered once daily [86]. Initially developed by GENFIT, Ipsen acquired global exclusive rights to elafibranor in 2021 [87,88,89] (Scheme 11).

The accelerated approval is predicated on the findings of a Phase II ELATIVE trial [90]. This multicenter, randomized, double-blind, placebo-controlled study evaluated the efficacy and safety of elafibranor at a dosage of 80 mg once daily in patients with PBC who were either insufficiently responsive to or intolerant of first-line therapy with UDCA. The results indicated that 51% of patients receiving Iqirvo achieved a cholestatic response compared to only 4% in the placebo group (*p* < 0.0001). The cholestatic response was defined as an alkaline phosphatase (ALP) level < 1.67 times the upper limit of normal (ULN), along with a ≥15% reduction in ALP at 52 weeks, and total bilirubin (TB) levels ≤ ULN. Both ALP and bilirubin are critical biomarkers for disease progression; their reduction implies diminished cholestatic injury and enhanced liver function. The first secondary endpoint—the normalization of ALP at 52 weeks—also demonstrated significant improvement with Iqirvo relative to placebo. Patients administered Iqirvo exhibited greater reductions in the PBC Worst Itch Numeric Rating Scale (NRS) score from baseline compared to those receiving placebo. Regarding safety, common adverse reactions included weight gain, abdominal pain, diarrhea, nausea, and vomiting. Some participants treated with Iqirvo reported myalgia, myopathy, rhabdomyolysis, fractures, drug-induced liver injury, allergic reactions, or biliary obstruction that could potentially affect fetal and neonatal development.

## 12. Sofdra (Sofpironium)

**Scheme 12:**
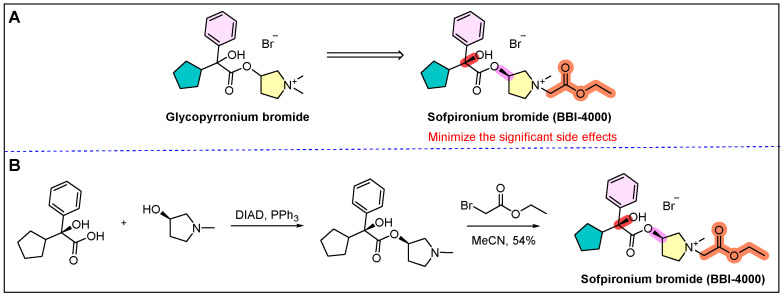
The discovery (**A**) and synthesis (**B**) of sofpironium.

Primary axillary hyperhidrosis is a condition characterized by excessive sweating that exceeds the physiological requirements for temperature regulation [91]. This debilitating disorder significantly affects patients’ quality of life, impacting work efficiency, daily activities, emotional well-being, and interpersonal relationships. It ranks as the third most prevalent skin disease in the United States (following acne and atopic dermatitis), with approximately 10 million individuals affected by primary axillary hyperhidrosis. On 18 June 2024, the U.S. FDA approved Sofdra (sofpironium) 12.45% Topical Gel for the treatment of primary axillary hyperhidrosis in children aged nine years and older as well as adults [92]. Sofdra represents the first new chemical entity to receive FDA approval for this condition, providing a safe and effective therapeutic option for patients who have limited alternatives [93]. Sofpironium functions as an anticholinergic agent that mitigates excessive sweating by inhibiting acetylcholine release within sweat glands. It effectively modulates sympathetic nervous system signaling in these glands to reduce sweat production. The therapy administers sofpironium through a gel formulation applied to the underarm area using a proprietary applicator designed to prevent direct contact with hands. Sofpironium bromide, a glycopyrronium derivative, has a modified structure that enables rapid hydrolytic inactivation [94,95] (Scheme 12). This change greatly minimizes the side effects typically linked to traditional anticholinergic drugs.

The FDA approval of Sofdra is predicated on two pivotal Phase III studies, termed “CARDIGAN,” which evaluated its efficacy and safety in a cohort of 701 patients diagnosed with primary axillary hyperhidrosis [96,97]. Participants were randomly assigned to receive either sofpironium gel or a placebo, administered nightly for a duration of six weeks. In Cardigan I, the initial average score of Hyperhidrosis Disease Severity Measure-Axillary (HDSM-Ax) was 3.5. The median sweat production within five minutes (GSP) was measured at 214.1 mg for the sofpironium group compared to 228.6 mg for the placebo group. Results indicated that 49% of patients treated with sofpironium experienced an improvement in their HDSM-Ax scores by at least two points by day 43, in contrast to only 29% in the placebo group (treatment difference: 18% [95% CI, 8–29]). Furthermore, the sofpironium group demonstrated a more significant change in GSP from baseline (−128 vs. −100). In the Cardigan II study, the initial average HDSM-Ax score was slightly elevated at 3.6. The median GSPs were 207.7 mg for sofpironium and 231.1 mg for the placebo at the beginning of the study. In this study, results revealed that 64% of patients receiving sofpironium improved their HDSM-Ax scores by at least two points by day 43 compared to 48% in the placebo cohort (treatment difference: 17% [95% CI, 6–27]). The change in GSP also favored those receiving sofpironium (−143 vs.−134). Common adverse reactions associated with sofpironium include dry mouth, blurred vision, pain and erythema, pupil dilation, and irritation occurring at the site of application.

## 13. Ohtuvayre (Ensifentrine)

**Scheme 13:**
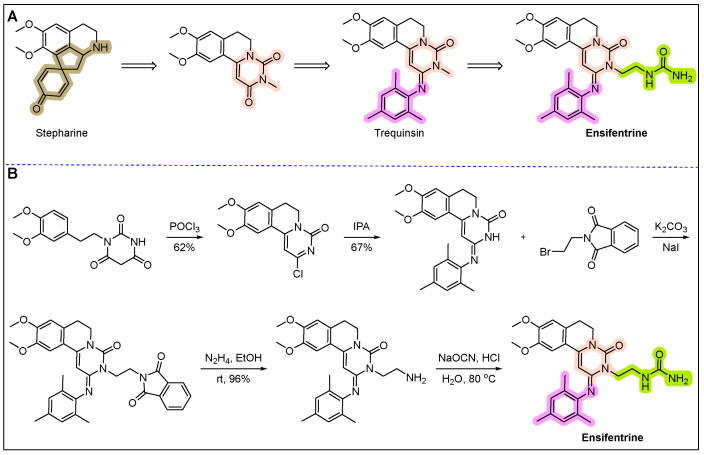
The discovery (**A**) and synthesis (**B**) of ensifentrine.

Chronic obstructive pulmonary disease (COPD) encompasses various lung conditions, including chronic bronchitis and emphysema, which restrict airflow and complicate the breathing process [98]. Patients frequently continue to experience symptoms despite the optimal utilization of existing therapies, underscoring the necessity for novel treatments that effectively alleviate symptoms, dilate bronchi, and mitigate disease progression. Phosphodiesterase 3 (PDE3) and Phosphodiesterase 4 (PDE4) inhibitors play a significant role in modulating diverse respiratory functions at both cellular and tissue levels. PDE3 regulates the concentrations of cyclic adenosine monophosphate (cAMP) and cyclic guanosine monophosphate (cGMP) within airway smooth muscle, thereby controlling bronchial tone. In contrast, PDE4 modulates cAMP levels in airway inflammatory cells while also activating these cells. Research has demonstrated that PDE4 inhibitors can stimulate the cystic fibrosis transmembrane conductance regulator (CFTR), leading to an enhancement in ciliary beat frequency within human bronchial epithelial cells. Given that both PDE isoenzymes are concurrently present on inflammatory cells as well as airway smooth muscle, their combined inhibition may produce additive or synergistic anti-inflammatory effects alongside bronchodilation benefits. On 26 June 2024, the U.S. FDA granted approval for Ohtuvayre (ensifentrine) as a maintenance treatment for adults diagnosed with chronic obstructive pulmonary disease (COPD) [99]. This milestone represents Verona Pharma’s first commercial product launch in over two decades and introduces an innovative inhalation therapy characterized by a novel mechanism of action. Ensifentrine is a unique dual inhibitor of phosphodiesterase type 3 (PDE3) and phosphodiesterase type 4 (PDE4), effectively combining bronchodilator and non-steroidal anti-inflammatory drug (NSAID) properties within a single compound [100]. It can be administered directly into the lungs using a standard jet nebulizer, which does not necessitate high inhalation airflow or intricate hand coordination. The dual inhibition mechanism offers a synergistic effect that enhances therapeutic outcomes related to bronchiectasis, airway inflammation, and mucosal ciliary clearance when compared to the isolated inhibition of either PDE3 or PDE4 alone. Natural products are an important source of inspiration for drug development [101,102,103,104,105,106,107,108,109,110,111,112,113,114,115]. Initial studies on synthesizing ensifentrine began with the structural modification of trequinsin derived from the natural stepharine [116,117] (Scheme 13).

The approval of ensifentrine is grounded in data derived from the Phase III ENHANCE Trial, a multicenter, randomized, double-blind, placebo-controlled study (NCT04542057) [118]. This trial comprised two components: ENHANCE-1, which included 760 patients, and ENHANCE-2 with 789 patients; 69% and 55% of participants were co-administered a long-acting muscarinic antagonist or a long-acting β2-receptor agonist, respectively. The results indicated that Ensifentrine significantly enhanced lung function across both trials and supported a reduction in exacerbation rates within a diverse COPD population. Ohtuvayre was well tolerated throughout both trials, with adverse event rates comparable between treatment groups. In Trial 1, adverse events occurred in 38.4% of Ohtuvayre patients compared to 36.4% for the placebo group; similarly, Trial 2 demonstrated analogous rates at 35.3% for Ohtuvayre versus 35.4% for placebo.

## 14. Leqselvi (Deuruxolitinib)

**Scheme 14:**
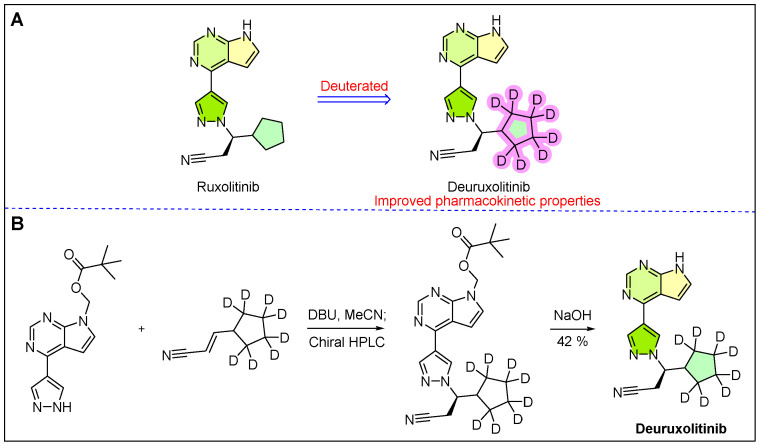
The discovery (**A**) and synthesis (**B**) of deuruxolitinib.

Alopecia areata is an autoimmune disorder characterized by the immune system’s erroneous attack on hair follicles, resulting in abrupt hair loss on the scalp, face, and occasionally other areas of the body [119]. The scalp is predominantly affected. This condition not only impacts physical appearance but also has significant psychological ramifications. Currently, treatment options for alopecia areata remain limited. On 25 July 2024, Sun Pharma’s oral JAK inhibitor Leqselvi (deuruxolitinib) received approval from the U.S. FDA for the treatment of severe alopecia areata [120]. The FDA granted Leqselvi both fast-track status and breakthrough therapy due to its notable efficacy and safety demonstrated in clinical trials. This approval provides patients with a novel therapeutic option aimed at promoting hair regrowth and improving quality of life. The oral administration of deuterated drugs typically exhibits pharmacokinetic advantages when compared to their non-deuterated counterparts [121,122]. Deuruxolitinib is a deuterated derivative of ruxolitinib. Its structural features enhance the stability of the five-membered ring, improving its pharmacokinetic properties [123,124] (Scheme 14).

Deuruxolitinib functions as an oral selective inhibitor of JAK1 and JAK2, exhibiting efficacy comparable to that of deuterorusotinib. The approval was granted based on data from two Phase III clinical trials, THRIVE-AA1 [NCT04518995] and THRIVE-AA2 [NCT04797650], involving a total of 1220 participants diagnosed with alopecia areata. Results indicated that the administration of an 8 mg dose resulted in a statistically significant reduction in hair loss severity score (SALT ≤ 20), indicating at least 80% scalp coverage compared to placebo as early as week eight; this effect was sustained throughout the duration of the study [125,126]. At week 24, approximately 46% of participants achieved over 80% scalp coverage, with up to 20% experiencing nearly complete recovery of their scalp hair (coverage ≥ 95%). The most frequently reported adverse reactions among patients receiving LEQSELVI 8 mg included headache, acne, and nasopharyngitis.

## 15. Voranigo (Vorasidenib)

**Scheme 15:**
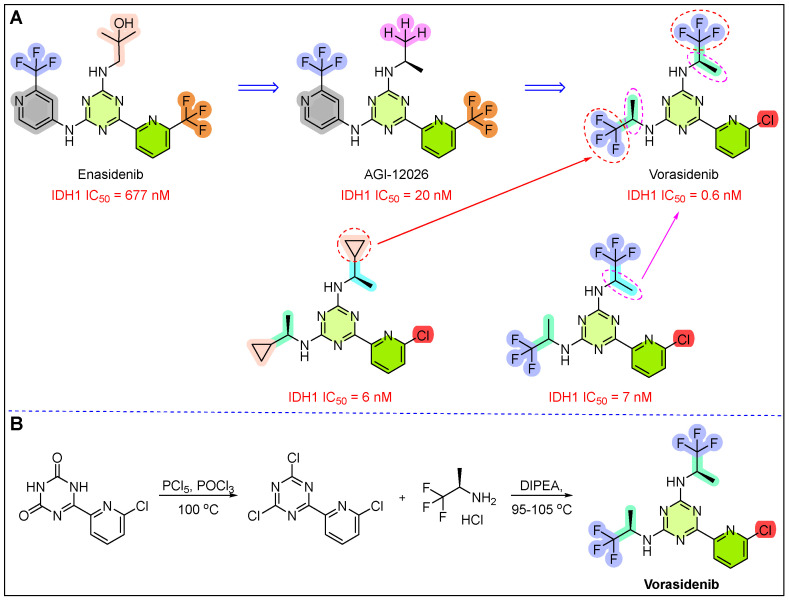
The discovery (**A**) and synthesis (**B**) of vorasidenib.

Glioma is a type of brain cancer that significantly affects brain function and manifests various symptoms [127]. Among adults under the age of 50, diffuse gliomas with isocitrate dehydrogenase (IDH) mutations represent the most prevalent malignant primary brain tumors. Current treatment modalities are unable to cure this disease. On 6 August 2024, the U.S. FDA granted approval for Voranigo (vorasidenib), developed by Servier Pharmaceuticals, for postoperative treatment in adults and children aged 12 years and older diagnosed with grade 2 astrocytoma or oligodendroglioma harboring susceptible IDH1 or IDH2 mutations [128]. As critical targets in oncological therapy, IDH1 and IDH2 play significant roles in tumor metabolic pathways. Voranigo functions as an oral dual inhibitor that selectively targets mutated IDH1/2 proteins to inhibit their activity, thereby attenuating cancer cell proliferation. Notably, Voranigo is the first targeted therapy approved by the U.S. FDA specifically for grade 2 IDH-mutant glioma and has been designated as a breakthrough therapy [129]. The structure–activity relationship studies showed that trifluoromethyl substitution had significant inhibitory activity, with an IC_50_ value of 0.6 nM [130] (Scheme 15).

The approval was predicated on findings from the INDIGO trial—a randomized, double-blind, placebo-controlled Phase III study involving 331 patients who were assigned to receive either a daily dose of 40 mg of Voranigo or a placebo until disease progression or unacceptable toxicity occurred [131]. Patients within the placebo group had the option to switch to Voranigo upon the radiological confirmation of disease progression. Results demonstrated that PFS was significantly prolonged among those receiving Voranigo at an average of 27.7 months compared to just 11.1 months for those receiving placebo; this resulted in a reduced risk of disease progression or death by approximately 61% (HR = 0.39; 95% CI: 0.27–0.56; *p* < 0.0001). The most frequently reported adverse reactions (≥15%) included fatigue, headache, COVID-19, musculoskeletal pain, diarrhea, nausea, and seizures. Voranigo significantly prolonged progression-free survival in patients, demonstrating notable efficacy and favorable tolerability compared to placebo. Its approval marks a significant advancement in the treatment of glioma, providing new hope for patients and their families confronting this challenging disease.

## 16. Livdelzi (Seladelpar)

**Scheme 16:**
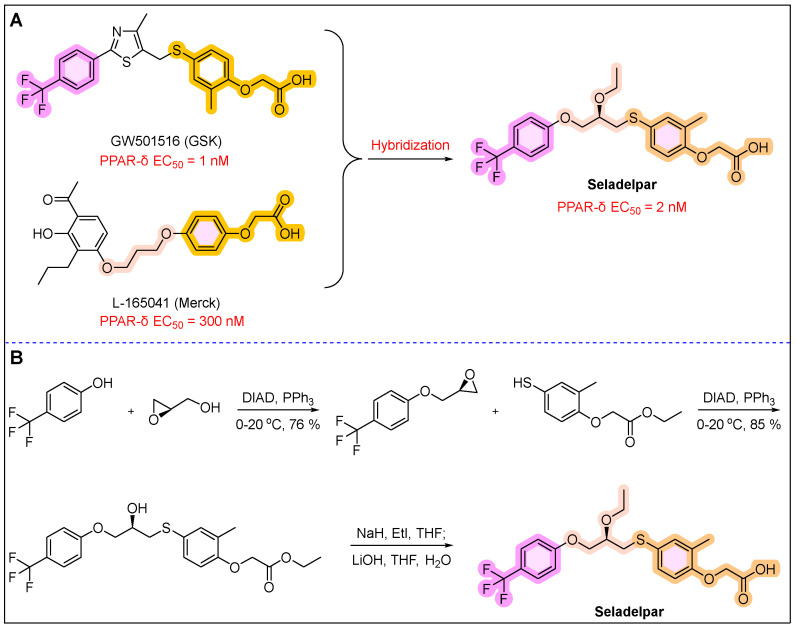
The discovery (**A**) and synthesis (**B**) of seladelpar.

Cholestatic hepatitis is a rare chronic autoimmune disease affecting the bile ducts, which can result in liver damage and potentially lead to liver failure if left untreated [132]. Currently, there is no definitive cure for this condition. On August 14, the U.S. FDA granted accelerated approval for Gilead Sciences’ oral PPAR-δ agonist Livdelzi (seladelpar) [133]. This medication may be used in conjunction with ursodeoxycholic acid (UDCA) for adults diagnosed with primary biliary cholangitis (PBC) who do not respond adequately to UDCA or as monotherapy for those who are intolerant to it. Livdelzi represents a novel therapeutic approach for liver diseases and signifies a substantial advancement in the treatment of PBC. Clinical trials have demonstrated that Livdelzi exhibits sustained efficacy and safety, including the normalization of alkaline phosphatase (ALP) levels in certain patients during the studies conducted [134]. Notably, Livdelzi was acquired through an investment in CymaBay Therapeutics rather than being developed internally by Gilead. CymaBay identified that the PPAR-δ agonist GW501516 has a dual effect on systemic circulation in healthy men, indicating its potential for treating dyslipidemia, obesity, and diabetes. Seladelpar was then developed by modifying existing GW501516 (GSK) and L-165041 (Merck) using molecular hybridization strategies [135,136] (Scheme 16).

The accelerated approval was primarily based on data derived from the placebo-controlled Phase III RESPONSE study, wherein 62% of patients receiving Livdelzi achieved the primary endpoint of composite biochemical response at 12 months compared to only 20% among those on placebo [137]. Furthermore, following treatment with Livdelzi, 25% of participants had normalized ALP levels at the 12-month mark—an important biomarker indicative of risks associated with cholestasis—while no such changes were observed within the placebo group. A significant secondary endpoint involved pruritus scores; patients treated with Livdelzi experienced notable reductions in symptoms relative to those receiving placebo. The FDA’s decision was influenced by these reductions in ALP levels; however, improvements concerning survival rates or the prevention of liver decompensation events remain unconfirmed at this time. The continued approval of Livdelzi for its current indications may depend on confirmatory trials that validate its clinical efficacy.

## 17. Rybrevant (Amivantamab)

**Scheme 17:**
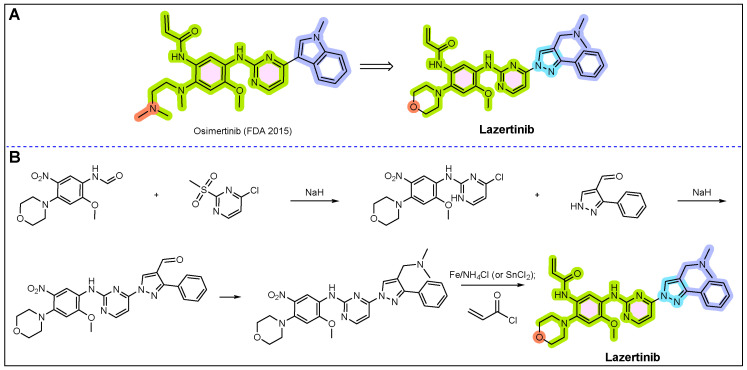
The discovery (**A**) and synthesis (**B**) of lazertinib.

Lung cancer is the most diagnosed malignancy and the leading cause of cancer-related deaths worldwide [138,139,140,141,142,143]. The most prevalent driver mutations in non-small cell lung cancer (NSCLC) are associated with alterations in the epidermal growth factor receptor (EGFR) gene, which encodes a receptor tyrosine kinase that plays a critical role in regulating cellular growth and division [144,145,146]. Among patients exhibiting EGFR alterations, approximately 90% of cases can be attributed to exon 19 deletions and the L858R mutation located in exon 21; insertion mutations within exon 20 represent the third most common category [147]. On 19 August 2024, the U.S. FDA granted approval for the combination therapy of Rybrevant (amivantamab), a bispecific antibody developed by Johnson & Johnson, alongside Lazcluze (lazertinib), a third-generation EGFR tyrosine kinase inhibitor (TKI) [148]. This therapeutic regimen is indicated for the first-line treatment of adult patients diagnosed with locally advanced or metastatic NSCLC harboring either EGFR exon 19 deletions or L858R substitutions within exon 21. Rybrevant is a humanized bispecific antibody targeting both EGFR and MET receptors, effectively blocking signaling through these pathways while directing immune cells to target tumors characterized by activating and resistant EGFR/MET mutations. Lazcluze is an oral TKI known for its high selectivity and ability to penetrate the blood–brain barrier. As the inaugural regimen available in the United States that offers extended progression-free survival compared to osimertinib without necessitating chemotherapy, Rybrevant combined with Lazcluze represents a novel first-line treatment option for patients with NSCLC. The design inspiration for lazertinib (YH25448) may have originated from osimertinib [149,150] (Scheme 17).

The approval of this combination therapy was based on the Phase III MARIPOSA trial (NCT04487080), which enrolled 1074 patients with locally advanced or metastatic non-small cell lung cancer (NSCLC) harboring exon 19 deletions or exon 21 L858R mutations who had not received prior systemic treatment [151]. Patients were randomized in a ratio of 2:2:1 to receive either lazertinib plus amivantamab, osimertinib monotherapy, or lazertinib monotherapy until disease progression or unacceptable toxicity occurred. The primary efficacy endpoint was PFS, assessed by blinded independent central review (BICR) for comparison between the lazertinib and amivantamab group versus osimertinib. Overall survival (OS) served as a key secondary endpoint. In comparison to osimertinib, the PFS for the combination of lazertinib and amivantamab demonstrated a statistically significant improvement, reducing the risk of disease progression or death by 30%. The median PFS for the combination group was reported at 23.7 months (95% CI: 19.1–27.7), whereas it was recorded at 16.6 months (95% CI: 14.8–18.5) for the osimertinib group. Common adverse reactions occurring in ≥20% of patients included rash, nail toxicity, musculoskeletal pain, edema, oral mucositis, skin dryness, as well as itching, nausea, and ocular toxicity.

## 18. Miplyffa (Arimoclomol)

**Scheme 18:**
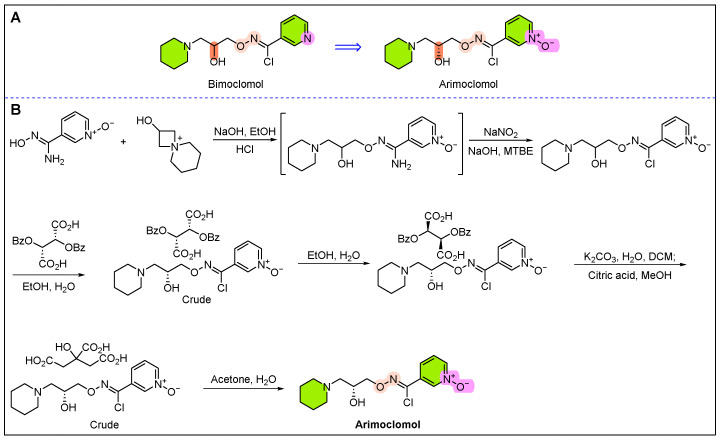
The discovery (**A**) and synthesis (**B**) of arimoclomol.

Niemann–Pick disease type C (NPC) is a rare, progressive neurodegenerative lysosomal storage disorder caused by mutations in the NPC1 or NPC2 genes [152]. These mutations lead to impaired cholesterol and lipid transport within cells, resulting in the accumulation of these substances, particularly within neurons. Patients with NPC may experience significant physical and cognitive limitations that affect various functions such as speech, cognition, swallowing, ambulation, and fine motor skills. The diagnostic process can span several years; moreover, the condition is irreversible and often leads to premature mortality. On 20 September 2024, the U.S. FDA granted approval for Miplyffa (arimoclomol), developed by Zevra Therapeutics, marking it as the first treatment specifically indicated for Niemann–Pick disease type C [153]. Miplyffa is recommended for use in conjunction with miglustat to address neurological symptoms associated with NPC in both adults and children aged two years and older. Bimoclomol and arimoclomol have been identified as co-inducers of heat shock proteins [154,155] (Scheme 18).

The approval of Miplyffa is based on data from a Phase II/III trial (ClinicalTrials.gov identifier: NCT02612129) involving diagnosed patients with NPC aged between 2 and 19 years (*N* = 50) [156]. Participants were randomly assigned in a 2:1 ratio to receive oral arimoclomol or placebo three times daily. The primary endpoint was the change from baseline in the four-domain NPC clinical severity score (R4DNPCCSS), which assesses disease progression through walking, speech, swallowing, and fine motor skills. Results at the 12-month mark indicated that the change in R4DNPCCSS from baseline demonstrated that arimoclomol treatment significantly slowed NPC progression compared to placebo (76% in the arimoclomol group vs. 81% in the placebo group; treatment difference −2.2 [95% CI, −3.8, −0.6]). Common adverse reactions included upper respiratory tract infections, diarrhea, and weight loss.

## 19. Aqneursa (Levacetylleucine)

**Scheme 19:**
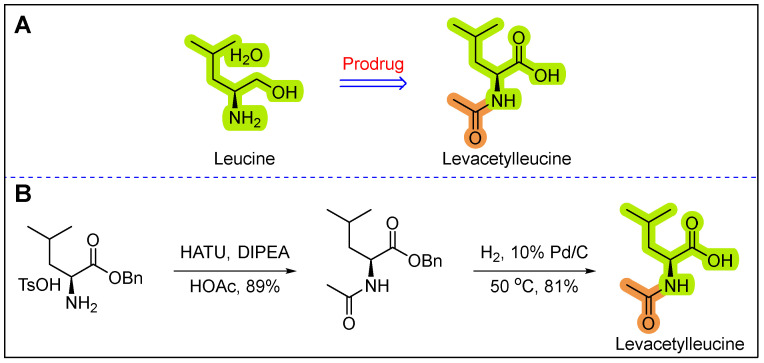
The discovery (**A**) and synthesis (**B**) of levacetylleucine.

On 24 September 2024, the U.S. FDA granted approval for Aqneursa (levacetylleucine), a therapeutic agent developed by IntraBio, aimed at addressing neurological symptoms associated with NPC in both adult and pediatric patients weighing at least 15 kg [157]. This marks the second NPC therapy to receive FDA approval within a week following the announcement of Miplyffa (arimoclomol) by Zevra Therapeutics. Aqneursa is characterized as a modified amino acid that effectively traverses the blood–brain barrier through monocarboxylic acid transporters, thereby facilitating optimal drug distribution to target tissues. It has been shown to restore mitochondrial and lysosomal functions within cells while enhancing glucose metabolism in the cerebellum, ultimately improving cerebellar activity. The FDA designated this therapy with orphan drug status, rare pediatric disease designation, and priority review status. Prodrugs are advanced derivatives of therapeutic agents designed to improve the drug’s pharmacokinetic profile [158,159,160]. First identified in 1901, levacetylleucine, an acetylated derivative (prodrug) of leucine, is used in its racemic form to treat vertigo [161,162] (Scheme 19).

The approval of Aqneursa was predicated on data derived from a pivotal phase IB1001-301 trial (NCT05163288), which demonstrated that it successfully achieved its primary endpoint: an improvement in Scale for the Assessment and Rating of Ataxia (SARA) scores alongside the functional SARA (fSARA) [163]. Furthermore, the therapy met all key secondary endpoints including the Spinocerebellar Ataxia Functional Index (SCAFI), Modified Disability Assessment Scale, and EuroQuol-5 Dimensions (EQ-5D) quality of life scale. Results published in the *New England Journal of Medicine* indicated that patients receiving levacetylleucine exhibited greater improvements in fSARA scores compared to those administered placebo (−0.4 average treatment difference). After 12 weeks of treatment, total SARA scores changed by −1.97 ± 2.43 points for the treatment group versus −0.60 ± 2.39 points for the placebo group; moreover, levacetylleucine was well tolerated with comparable adverse event rates observed between groups. The most frequently reported adverse events included abdominal pain, dysphagia, upper respiratory infections, and vomiting. The most common adverse events included abdominal pain, difficulty swallowing, upper respiratory tract infections, and vomiting.

## 20. Cobenfy (Xanomeline/Trospium Chloride)

Schizophrenia is a complex mental illness that presents significant challenges in the field of mental health [164]. On 26 September 2024, the U.S. FDA granted approval for Cobenfy (KarXT), a combination of xanomeline and trospium chloride developed by Bristol Myers Squibb, for the treatment of schizophrenia [165]. This marks the first antipsychotic medication designed to specifically target cholinergic receptors. Cobenfy integrates xanomeline and trospium chloride to activate muscarinic acetylcholine receptors in the brain while minimizing activity at peripheral receptors. By stimulating M1 and M4 muscarinic receptors, xanomeline effectively alleviates negative symptoms associated with schizophrenia, enhances cognitive performance, and addresses other psychiatric issues such as hallucinations and delusions [165]. Trospium chloride functions as a muscarinic receptor antagonist to mitigate potential peripheral side effects induced by xanomeline. Historically, treatments for schizophrenia have predominantly focused on dopamine receptor pathways; thus, this approval signifies a novel mechanistic approach after decades of reliance on traditional therapies.

Bristol Myers Squibb has reported positive top-line results from two open-label Phase III trials: EMERGENT-4 and EMERGENT-5 [166]. In adult patients diagnosed with schizophrenia, symptoms demonstrated continued improvement following 52 weeks of treatment with Cobenfy (xanomeline combined with trospium chloride), indicating sustained efficacy over time.

## 21. Itovebi (Inavolisib)

On 10 October 2024, the U.S. FDA approved Genentech’s Itovebi (inavolisib) for use with Ibrance (palbociclib) and Fulvestrant in treating adult patients with locally advanced or metastatic breast cancer that is hormone receptor (HR)-positive, HER2-negative, and has a PIK3CA mutation, who have developed resistance to endocrine therapy [167]. Itovebi (inavolisib) is a PI3K inhibitor targeting the PI3Kα subtype. It inhibits the PI3K signaling pathway to block tumor cell growth and proliferation. In HR-positive breast cancer, the dysregulation of this pathway due to PIK3CA mutations often leads to resistance against standard endocrine therapy. The high efficacy and specificity of inavolisib aim to reduce the burden and toxicity associated with other PI3K inhibitors.

**Scheme 20:**
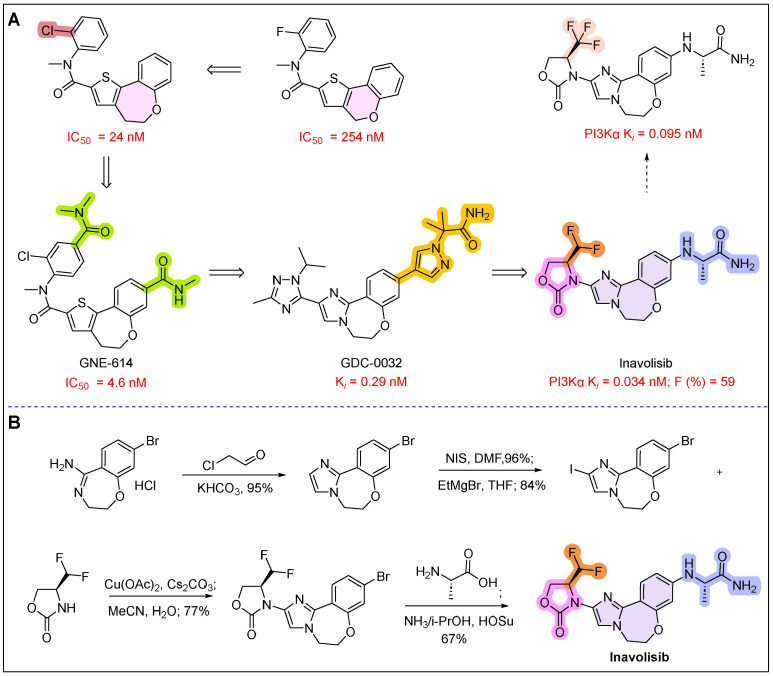
The discovery (**A**) and synthesis (**B**) of inavolisib.

The researchers initially employed a ring expansion strategy to obtain a seven-membered ring compound, which was further optimized and synthesized, resulting in toxic byproducts like aniline. They then replaced the amide structure using bioelectronic strategies, optimizing side chains and substituting thiophene rings while considering selectivity and metabolic stability. Finally, they refined the triazole segments and side chains to develop the drug [168,169] (Scheme 20).

This approval is based on the randomized, double-blind, placebo-controlled multicenter trial INAVO120 (NCT04191499), which included 325 breast cancer patients who experienced disease progression during adjuvant endocrine therapy or within 12 months after treatment completion and had not received prior systemic treatment for locally advanced or metastatic disease [170]. The INAVO120 trial results showed that patients receiving inavolisib in combination had a significantly lower risk of disease progression or death compared to those receiving only palbociclib and Fulvestrant (placebo group). The median PFS was 15.0 months (95% CI: 11.3–20.5) for the inavolisib group versus 7.3 months (95% CI: 5.6–9.3) for the placebo group, with a hazard ratio (HR) of 0.43 (95% CI: 0.32–0.59) and a *p*-value < 0.0001, indicating significant efficacy. Additionally, the objective response rate (ORR) was 58% (95% CI: 50–66) in the inavolisib group compared to 25% (95% CI: 19–32) in the placebo group, with median durations of response (DORs) at 18.4 months (95% CI: 10.4–22.2) and 9.6 months (95% CI: 7.4–16.6), respectively. Although interim overall survival analysis did not reach statistical significance, there was an overall benefit observed for the inavolisib group with an HR of 0.64 (95% CI: 0.43–0.97). Common adverse reactions associated with inavolisib (>20%) include neutropenia, anemia, elevated fasting blood glucose, thrombocytopenia, lymphopenia, stomatitis, diarrhea, hypocalcemia, fatigue, hypokalemia, increased creatinine levels, elevated alanine aminotransferase (ALT), nausea, hyponatremia, hypomagnesemia, rash, decreased appetite, COVID-19 infection, and headache.

## 22. Orlynvah (Sulopenem Etzadroxil and Probenecid)

Some patients with urinary tract infections (UTIs) are infected by bacterial strains that exhibit resistance to traditional antibiotics such as amoxicillin and ciprofloxacin, highlighting the urgent need for novel treatment options. On 25 October 2024, the U.S. FDA granted approval to Iterum Therapeutics’ Orlynvah (sulopenem etzadroxil and probenecid) for the treatment of uncomplicated UTIs in adult women caused by specific microorganisms—namely, *Escherichia coli*, *Klebsiella pneumoniae*, or *Proteus mirabilis*—where few or no alternative oral antimicrobial therapies are available [8]. Orlynvah represents the first oral carbapenem antibiotic approved by the FDA and combines sulopenem etzadroxil (a carbapenem antibiotic) with probenecid (a renal tubular transporter inhibitor). Furthermore, it is noteworthy that this marks only the second UTI treatment drug approved by the FDA in two decades, providing an important alternative to existing antibiotics. In vitro studies have demonstrated its efficacy against Gram-negative, Gram-positive, and anaerobic bacteria resistant to other antimicrobial agents.

The FDA’s approval of sulopenem etzadroxil and probenecid was based on data derived from two pivotal Phase III trials: SURE 1 (NCT03354598) and REASSURE (NCT05584657). The SURE 1 trial evaluated the safety and efficacy of sulopenem etzadroxil combined with probenecid compared to ciprofloxacin in treating uncomplicated UTIs among a cohort of 1660 adult women. This treatment exhibited a response rate of 48% in patients with ciprofloxacin-resistant infections versus a response rate of 33% for those treated with ciprofloxacin alone. The REASSURE trial compared this combination therapy against amoxicillin/clavulanate (Augmentin), involving a study population of 2214 adult women diagnosed with uncomplicated UTIs. Sulopenem etzadroxil combined with probenecid achieved a response rate of 62% against pathogens sensitive to amoxicillin/clavulanate; conversely, the group receiving amoxicillin/clavulanate had a response rate of only 55%. Both clinical trials indicated favorable tolerability profiles for Orlynvah across all participants involved; specifically, a total of 1932 patients were treated throughout both SURE 1 and REASSURE studies.

## 23. Revuforj (Revumenib)

**Scheme 21:**
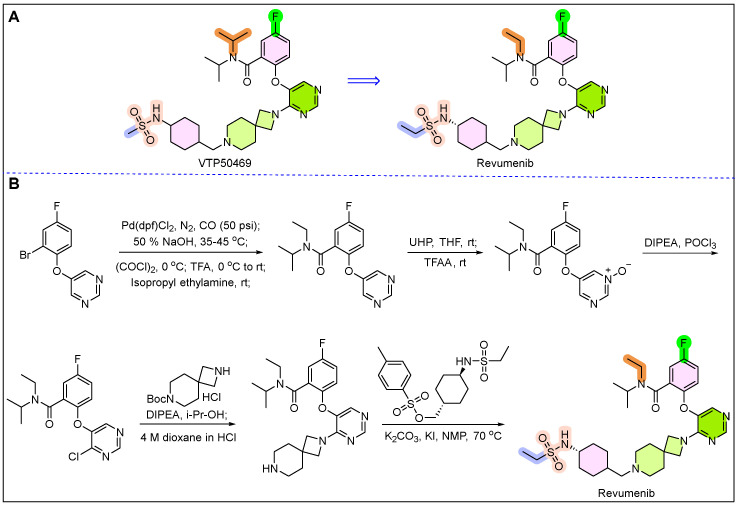
The discovery (**A**) and synthesis (**B**) of revumenib.

KMT2A gene rearrangement (KMT2Ar) is associated with a type of malignant acute leukemia that has a poor prognosis and high relapse rate. Over 95% of patients with KMT2Ar acute leukemia exhibit KMT2A translocations, which occur when part of a chromosome breaks and fuses with another chromosome [171]. Menin, a scaffold protein, binds to KMT2A, activating leukemogenic genes like HOX and MEIS1. On 15 November 2024, the U.S. FDA approved revumenib (SNDX-5613, brand name: Revuforj), developed by Syndax Pharmaceuticals, as the first menin inhibitor for treating relapsed or refractory (R/R) acute leukemia in adults and pediatric patients aged one year and older with KMT2A translocations [172]. The binding of KMT2A fusion proteins to menin plays a crucial role in activating transcription pathways involved in KMT2Ar acute leukemia. Revumenib disrupts the interaction between wild-type KMT2A and fusion proteins by entering the menin binding pocket, thereby inhibiting HOX and MEIS gene expression and halting leukemia cell growth. Revumenib with significantly improved pharmacokinetic properties was obtained by replacing isopropylphenyl with ethyl [173,174] (Scheme 21).

The efficacy of Revuforj was assessed in a single-arm, open-label, multicenter trial (Study SNDX-5613-0700, NCT04065399; AUGMENT-101) involving 104 adult and pediatric patients with relapsed or refractory acute leukemia and KMT2A translocations [175]. Results indicated that the combined rate of complete response (CR) and complete response with incomplete hematologic recovery (CRh) was 21.2% (95% CI, 13.8–30.3), with a median duration of CR plus CRh lasting 6.4 months (95% CI, 2.7–not estimable). Among these patients, 13 achieved CR (12.5%), with a median duration of 4.3 months (95% CI, 1-not estimable), while 9 achieved CRh (8.7%), with a median duration of 6.4 months (95% CI, 1.9–not estimable). Common adverse reactions occurring in ≥20% included laboratory abnormalities, bleeding, and musculoskeletal pain. The significant clinical benefits and robust efficacy of Revumenib compared to previous therapies represent a major advancement and may become an important new treatment option for these patients.

## 24. Rapiblyk (Landiolol)

**Scheme 22:**
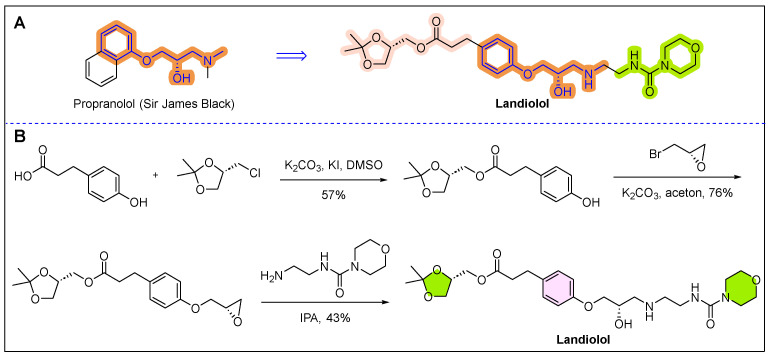
The discovery (**A**) and synthesis (**B**) of landiolol.

Supraventricular tachycardia, which includes conditions such as atrial fibrillation and atrial flutter, can manifest in patients with or without underlying heart disease. Given its potential impact on cardiac function and the associated risk of acute cardiovascular complications, immediate medical intervention is essential. On 22 November 2024, the U.S. FDA granted approval for Rapiblyk (landiolol), developed by AOP Health, specifically for the treatment of supraventricular tachycardia—including atrial fibrillation and atrial flutter—in critical care settings within hospitals [8]. Clinical study results indicate that Rapiblyk effectively controls heart rate rapidly while minimizing adverse effects on blood pressure [176]. The incorporation of chiral dioxolane into the phenyl group as well as piperazine into the amino group of propranolol led to the discovery of landiolol [177,178] (Scheme 22).

Rapiblyk is administered intravenously and functions as an ultra-short-acting adrenergic receptor antagonist with a β1/β2 selectivity ratio of 255. It is characterized by a rapid onset of action that allows for a swift reduction in heart rate without significantly lowering patients’ blood pressure. This medication is intended for use in emergency situations within cardiac intensive care units, operating rooms, and other critical care environments; it is suitable for short-term treatment under critical conditions.

## 25. Attruby (Acoramidis)

**Scheme 23:**
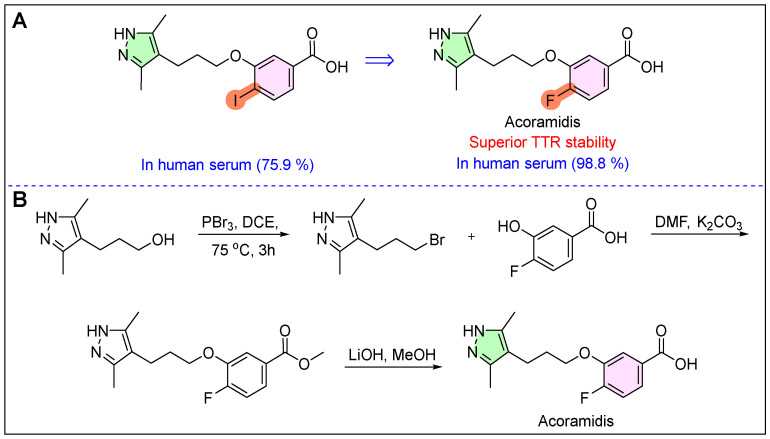
The structure (**A**) and synthesis (**B**) of acoramidis.

The characteristic of transthyretin amyloidosis (ATTR) is the formation of amyloid fibril deposits in tissues and organs, particularly affecting the heart and leading to cardiomyopathy. Both Attruby and tafamidis (tafamidis meglumine) are designed to stabilize the misfolded TTR protein associated with this disease. Tafamidis has been approved in the United States since 2019 for treating transthyretin-mediated amyloid cardiomyopathy (ATTR-CM) and managing ATTR-related polyneuropathy [179]. Attruby received approval in November 2024 as a new therapy for TTR stabilization, making it the first drug labeled for the near-complete stabilization of TTR [8]. This medication maintains TTR’s natural functions as a transporter of thyroid hormone and vitamin A while also showing beneficial effects on cardiovascular outcomes. Here, TTR refers to transthyretin protein. The fluorine atom may enhance the interaction between acoramidis and TTR, improving its stabilizing efficacy towards TTR [180,181] (Scheme 23).

Safety data from a 30-month randomized, double-blind, placebo-controlled trial involving 421 ATTR-CM patients show that Attruby (712 mg orally twice daily) had a median exposure time of 29 months in safe populations [182]. Both the Attruby and placebo groups experienced higher rates of gastrointestinal adverse events, including diarrhea (11.6% vs. 7.6%) and upper abdominal pain (5.5% vs. 1.4%). Most events were mild and resolved without treatment discontinuation. Discontinuation due to adverse events was similar for both groups: 9.3% for Attruby and 8.5% for placebo.

## 26. Crenessity (Crinecerfont)

**Scheme 24:**
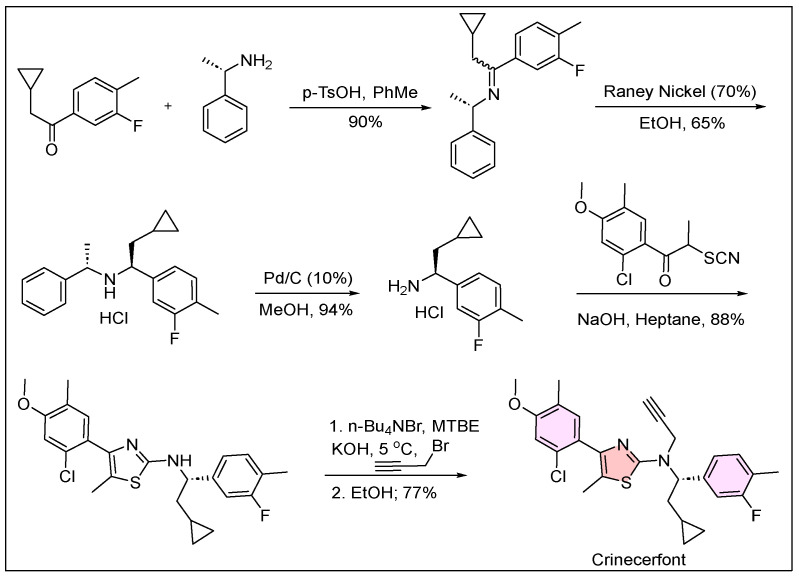
Synthesis of crinecerfont.

Classic congenital adrenal hyperplasia (CAH) is a rare, serious, and lifelong genetic disorder affecting the adrenal glands [183]. It impairs the ability of these glands to produce sufficient cortisol while leading to the excessive production of androgens, resulting in hormonal imbalance. On 13 December 2024, the U.S. FDA approved Crenessity (crinecerfont), developed by Neurocrine Biosciences, as an adjunct therapy used in combination with glucocorticoids for the treatment of classic congenital adrenal hyperplasia (CAH) in adults and pediatric patients aged four years and older [184]. Crinecerfont is an oral selective corticotropin-releasing factor 1 receptor (CRF1) antagonist that represents a “first-in-class” therapy for this severe endocrine disorder. It reduces and controls excessive adrenal androgen levels through a hormone-independent mechanism specifically targeting CAH caused by 21-hydroxylase deficiency. The synthesis of crinecerfont is illustrated in Scheme 24 [185].

According to published data from the Phase III CAHtalyst pediatric study, crinecerfont treatment resulted in a statistically significant reduction in serum androstenedione levels at week four compared to baseline (*p* = 0.0002), when compared to placebo [186]. This finding aligns with results from the Phase III CAHtalyst adult study, indicating that crinecerfont demonstrates efficacy across different age groups. At week twenty-eight, compared to placebo, crinecerfont treatment reduced the daily required dose of glucocorticoids while maintaining control over androgen levels (*p* < 0.0001). Furthermore, crinecerfont exhibited good safety and tolerability throughout the study period. The success of Crenessity not only lies in its capacity to effectively manage CAH symptoms but also reflects future directions within the pharmaceutical industry regarding chronic disease management: less reliance on hormones—enhanced quality of life.

## 27. Ensacove (Ensartinib)

**Scheme 25:**
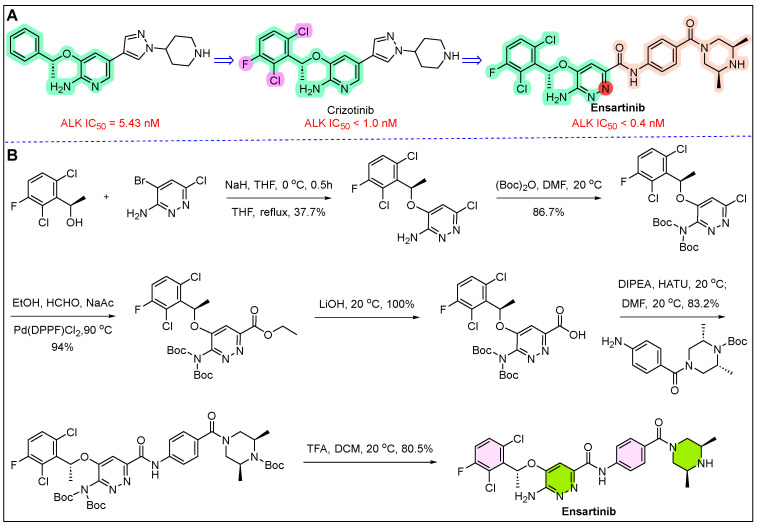
The discovery (**A**) and synthesis (**B**) of ensartinib.

Lung cancer is a leading cause of cancer-related deaths worldwide, especially non-small cell lung cancer (NSCLC), which comprises about 80–85% of cases [187,188,189]. ALK-positive tumors account for only 3–5% of NSCLC but represent a significant patient population with new cases each year [190]. Many ALK-positive metastatic NSCLC patients initially respond well to tyrosine kinase inhibitors (TKIs), but relapses are common. For those who have undergone second-generation ALK TKI therapy and whose condition worsens, treatment options become very limited. On 18 December 2024, the U.S. FDA approved ensartinib (X-396, brand name Ensacove, Xcovery Holdings, Inc.), an ALK inhibitor by Xcovery Holdings, for adults with locally advanced or metastatic ALK-positive NSCLC who have not received prior ALK inhibitor therapy [8]. Ensartinib overcame crizotinib-induced drug resistance by altering its structure [191,192] (Scheme 25).

The efficacy of Ensacove was evaluated in the eXALT3 study (NCT02767804), a randomized, controlled trial involving 290 ALK-positive NSCLC patients who had not received prior ALK-targeted therapy [193]. Patients were assigned in a 1:1 ratio to receive either Ensacove or Crizotinib. The primary endpoint was progression-free survival (PFS), assessed by independent central review. A key secondary endpoint was overall survival (OS). Ensacove showed a statistically significant improvement in PFS compared to Crizotinib, with a hazard ratio (HR) of 0.56 (95% CI: 0.40–0.79; *p*-value 0.0007). The median PFS for the Ensacove group was 25.8 months (95% CI: 21.8, not estimable), while, for the Crizotinib group, it was 12.7 months (95% CI: 9.2, 16.6). There was no significant difference in OS between groups (HR 0.88 [95% CI: 0.63, 1.23], *p*-value 0.4570). Common adverse reactions included rash, musculoskeletal pain, and constipation, each occurring in over 20% of patients. ENSACOVE is a novel and potent ALK inhibitor that effectively targets various ALK mutations in vitro experiments and offers new treatment options for ALK-positive NSCLC patients, potentially enhancing their survival and quality of life. This approval represents an important milestone for BeiGene globally.

## 28. Alyftrek (Vanzacaftor/Tezacaftor/Deutivacaftor)

**Scheme 26:**
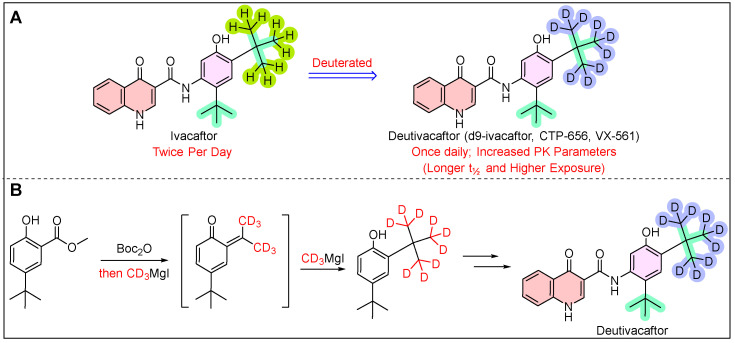
The discovery (**A**) and synthesis (**B**) of deutivacaftor.

Cystic fibrosis is a rare genetic disorder caused by defects in the CFTR protein due to mutations in the CFTR gene. Affected children inherit two defective CFTR genes, one from each parent. These defects impair salt and water flow in various organs, leading to thick mucus accumulation, chronic lung infections, and progressive lung damage [194]. On 20 December 2024, the U.S. FDA approved Alyftrek (vanzacaftor/tezacaftor/deutivacaftor), a next-generation combination of CFTR modulators developed by Vertex Pharmaceuticals for cystic fibrosis patients aged 6 years and older with at least one F508del mutation or other responsive mutations [194]. Vanzacaftor and tezacaftor bind to different sites on the CFTR protein, working together to enhance cellular processing and the transport of selected mutant forms like F508del-CFTR, increasing the amount of functional CFTR protein at the cell surface compared to either drug alone. Deutivacaftor improves the likelihood of opening CFTR channels on cell surfaces. The combined action of these three components increases both the quantity and functionality of CFTR at the cell surface, enhancing its activity as measured by chloride transport in vitro and sweat chloride levels in patients. Ivacaftor contains two tert-butyl segments, but its oxidative metabolism mainly occurs in one of them. This involves the CYP3A4-mediated hydroxylation of one methyl group, resulting in an alcohol that is further oxidized to carboxylic acids. The addition of D into the softer tert-butyl region acts as a deactivator (d9-ivactant, CTP-656, VX-561), while the other tert-butyl segment remains largely unchanged metabolically. Moreover, clinical studies show that d9-ivactant can be taken once daily at a single dose of 150 mg, compared to ivacaftor, which requires twice-daily dosing [195,196] (Scheme 26).

In two 52-week, randomized, double-blind, active-controlled trials (Trial 1, NCT05033080; Trial 2, NCT05076149), the efficacy of ALYFTREK and Trikafta was evaluated in 971 patients aged 12 years and older with cystic fibrosis who had at least one F508del mutation or an adaptive CFTR gene mutation [197]. Trikafta is a fixed-dose combination drug containing elexacaftor, tezacaftor, and ivacaftor. In Trials 1 and 2, compared to Trikafta, ALYFTREK treatment resulted in a least-squares mean difference in absolute change in percent predicted FEV1 (ppFEV1) from baseline to week 24 of 0.2 percentage points (95% CI: −0.7, 1.1) and 0.2 percentage points (95% CI: −0.5, 0.9), respectively. Since the lower limit of the 95% CI for this difference was greater than −3.0 percentage points (the pre-specified non-inferiority margin), these results indicate that ALYFTREK is not inferior to Trikafta. Additionally, a Phase III study (NCT05422222) involving children aged 6–11 years demonstrated that ALYFTREK met safety as the primary endpoint. Secondary endpoints showed benefits through absolute changes in ppFEV1 and sweat chloride relative to baseline for this age group. ALYFTREK was generally well tolerated across all studies. The most common adverse reactions (≥5% incidence higher than Trikafta by ≥1%) included cough, nasopharyngitis, upper respiratory tract infections, headache, influenza, and fatigue.

## 29. Conclusions and Outlook

The 27 small-molecule drugs approved by the FDA in 2024 exemplify significant advancements in therapeutic innovation, spanning diverse clinical applications across cancer, rare diseases, infectious diseases, and chronic conditions. These approvals underscore the structural diversity of the approved drugs, which are characterized by unique and effective pharmacophores, offering a strong foundation for future drug discovery and innovation. The integration of novel synthetic methods with comprehensive clinical strategies has been instrumental in addressing unmet medical needs and enhancing patient outcomes. Moving forward, the continued application of innovative synthetic approaches and clinical development strategies will play a critical role in advancing global healthcare and addressing pressing medical challenges. Furthermore, the success of these drugs highlights the potential for future advancements in precision medicine, combination therapies, innovative drug delivery systems, and the integration of digital health technologies, all of which are poised to transform therapeutic development and improve patient care. The following are several insights into future drug development:

**Exploring new therapeutic areas and mechanisms:** Many approved drugs target rare, drug-resistant, or undertreated diseases, such as Duchenne muscular dystrophy, Niemann–Pick disease type C, and WHIM syndrome. Their success highlights the need for future research to focus on unmet medical needs, particularly in rare and refractory diseases. Some drugs utilize novel mechanisms of action, like specific signaling pathway targeting. This innovative approach inspires new drug design ideas and encourages the exploration of unique therapeutic strategies. Additionally, some drugs enhance efficacy and reduce drug resistance by targeting multiple pathways simultaneously. Future development can further investigate multi-target or multi-mechanism combination therapies for more comprehensive disease management.

**Optimize the R&D process and regulatory strategies:** Eight drugs have been designated as breakthrough therapies, reflecting the FDA’s commitment to expediting the development and approval of innovative medications. Researchers and pharmaceutical companies can enhance drug development and market entry by engaging with regulatory authorities early and utilizing policies like breakthrough therapies, fast track, and priority review. Optimizing clinical trial design in the future can shorten R&D timelines, lower costs, and expedite new drug availability. By harnessing technologies like artificial intelligence, machine learning, and big data analysis, drug discovery can be accelerated while predicting clinical outcomes and optimizing treatment plans [198,199,200,201]. The integration of these technologies will further enhance the efficiency and accuracy of drug development in the future.

**Improve the success rate and accessibility of drug development:** Some drugs have achieved precise treatment through biomarker detection, enhancing therapeutic effects while reducing side effects. In the future, precision medicine will remain a key focus in drug development, prompting researchers to create more personalized treatment plans. Innovative delivery systems like nanoparticles and liposomes have improved drug stability and targeting [202,203]. Future advancements in drug delivery technology are expected to lead to more effective and low-toxicity medications. Additionally, some drugs show promise in developing countries with limited resources for treating conditions such as urinary tract infections and *Staphylococcus aureus* blood infections. Moving forward, drug development should prioritize global health issues to ensure that new drugs are accessible and affordable.

**Exploration of synergistic effects:** Some drugs have significantly improved their therapeutic effects when used in combination with other treatment methods, such as chemotherapy and immunotherapy. In the future, the exploration of combined treatment strategies will help overcome the limitations of single drugs and provide more comprehensive treatment plans. By monitoring patients’ biomarkers and treatment responses in real time and dynamically adjusting drug dosages and regimens, the therapeutic effect can be further enhanced and side effects reduced. In the future, this individualized treatment model will become more common.

**Ensure equal emphasis on innovation and security:** As personalized medicine advances, drug development must prioritize ethical issues like gene therapy safety, patient privacy, and equitable treatment access. The FDA’s approval process should balance innovation with ensuring drug safety and efficacy. Regulatory authorities need to collaborate closely with researchers and pharmaceutical companies to create a flexible, science-based regulatory framework that keeps pace with rapidly evolving drug technologies.

## Figures and Tables

**Table 1:** Small-molecule drugs approved by the US FDA during 2024 (https://www.fda.gov/drugs/novel-drug-approvals-fda/novel-drug-approvals-2024, accessed on 1 January 2025).

No.	Approval Date	Brand Name (Company)	Active Ingredient (Chemical Structure)	Clinical Trials (Administration)	Indication (Mechanism)	Possible Side Effects
1	2/22/2024	Exblifep (Allecra Therapeutics)	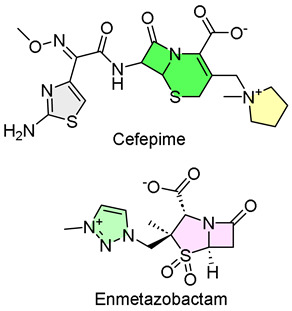	NCT03687255, composite response was achieved in 273 of 345 patients (intravenous, IV)	Complicated urinary tract infections (cephalosporin and β-lactamase inhibitor)	Allergic reactions, neurological issues, and diarrhea
2	3/14/2024	Rezdiffra (Madrigal Pharmaceuticals)	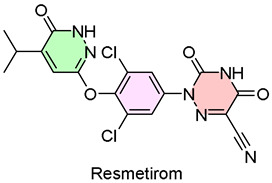	NCT03900429, resolving NASH and reducing liver scarring (oral administration)	Nonalcoholic steatohepatitis (thyroid hormone receptor β agonist)	Liver toxicity and inflammation of the gallbladder or pancreas
3	3/19/2024	Tryvio (Idorsia Pharmaceuticals)	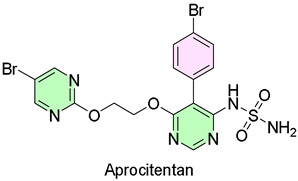	NCT03541174, lower sitting blood pressure (oral administration)	Hypertension (endothelin A and endothelin B receptors; antagonist)	Liver issues, harm to an unborn baby, and reduced sperm counts in males
4	3/21/2024	Duvyzat (Italfarmaco SpA)	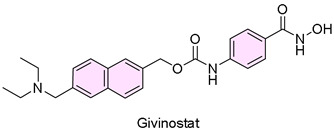	NCT02851797, experienced less decline in muscle function tests (oral administration)	Duchenne muscular dystrophy (histone deacetylase inhibitor)	Diarrhea, abdominal pain, low platelets, or high triglycerides
5	3/27/2024	Vafseo (Akebia Therapeutics)	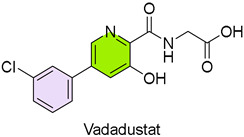	NCT02865850, NCT02892149, maintaining correct hemoglobin levels (oral administration)	Anemia due to chronic kidney disease (hypoxia-inducible factor prolyl hydroxylase [HIF-PH] inhibitor)	Thrombotic vascular events
6	3/29/2024	Voydeya (Alexion Pharmaceuticals)	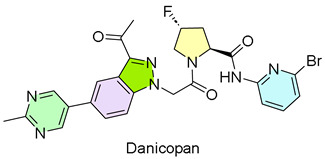	ALXN2040-PNH-301, Voydeya’s superiority when combined with ravulizumab or eculizumab	Extravascular hemolysis (factor D inhibitor)	Hepatic enzyme increases and hyperlipidemia
7	4/3/2024	Zevtera (Basilea Pharmaceutica)	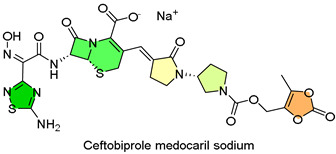	BPR-CS-009, BPR-CS-008, CAP-3001, BPR-PIP-002, overall success (IV)	*Staphylococcus aureus* bloodstream infections, ABSSSI and CABP; (cephalosporin antibiotic)	Nausea, headache, diarrhea, and fever
8	4/23/2024	Ojemda (Day One Biopharmaceuticals)	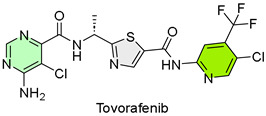	NCT04775485/FIREFLY-1, 51% of patients had complete, partial, or minor tumor shrinkage	Pediatric low-grade glioma (Pan-RAF kinase inhibitor)	Bleeding issues, skin reactions, liver issues, or stunted growth
9	4/26/2024	Xolremdi (X4 Pharmaceuticals)	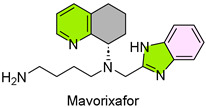	NCT03995108, more white blood cells and fewer infections	WHIM syndrome (CXCR4 antagonist)	Fetal harm, QTc interval prolongation
10	6/10/2024	Iqirvo (Ipsen biopharm)	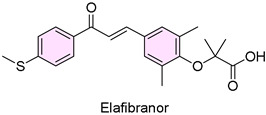	NCT04526665, more patients achieved biochemical response	Primary biliary cholangitis (PPARα/δ agonist)	Weight gain, diarrhea, and rash
11	6/18/2024	Sofdra (Botanix SB)	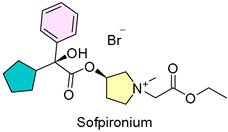	NCT03836287, NCT03948646, greater reduction in GSP (topical gel)	Primary axillary hyperhidrosis (anti-cholinergic agent)	Dry mouth, blurry vision, mydriasis
12	6/26/2024	Ohtuvayre (Verona Pharma)	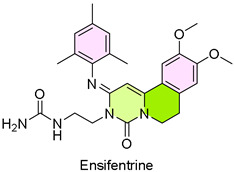	ENHANCE-1, ENHANCE-2, improved pulmonary function	Chronic obstructive pulmonary disease (phosphodiesterase 3/4 inhibitor)	Back pain, diarrhea, and mental health problems
13	7/25/2024	Leqselvi (Sun Pharma)	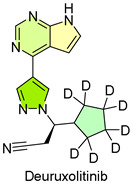	About 30% of subjects have 80% or more scalp hair	Severe alopecia areata (JAK1 and JAK2 inhibitor)	Serious infections, cardiovascular events, and immune system problems
14	8/6/2024	Voranigo (Servier Pharmaceuticals)	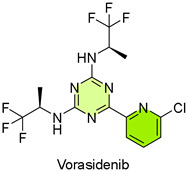	NCT04164901, improved overall PFS	IDH-mutant grade 2 glioma (IDH1 and IDH2 inhibitor)	Hepatoxicity and embryo-fetal toxicity
15	8/14/2024	Livdelzi (CymaBay Therapeutics)	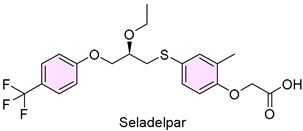	NCT04620733, more patients achieved biochemical response	Primary biliary cholangitis (peroxisome proliferator-activated receptor-δ (PPARδ) agonist)	Headaches, abdominal pain, and dizziness
16	8/19/2024	Lazcluze (Johnson & Johnson)	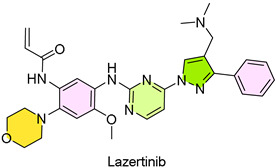	NCT04487080, median PFS was 23.7 months	Non-small-cell lung cancer (EGFR kinase inhibitor)	Lung, skin, and eye problems; blood clots
17	9/20/2024	Miplyffa (Zevra Therapeutics)	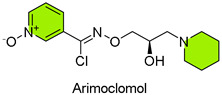	Less worsening of symptoms	Niemann–Pick disease type C	Hypersensitivity reactions, diarrhea
18	9/24/2024	Aqneursa (IntraBio)	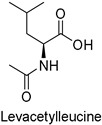	Less worsening of symptoms	Niemann–Pick disease type C	Abdominal pain, dysphagia, and vomiting
19	9/26/2024	Cobenfy (Bristol Myers Squibb)	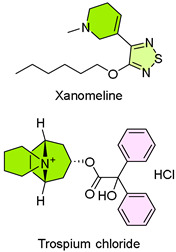	Meaningful reduction in symptoms	Schizophrenia (M1/M4 mAChR modulator)	Urinary retention, increased heart rate, decreased gastric motility, or facial and lip angioedema
20	10/10/2024	Itovebi (Genentech)	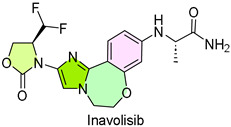	NCT04191499, the median progression-free survival (PFS) was 15.0 months	Advanced or metastatic breast cancer (mutant PI3Kα inhibitor and degrader)	Neutropenia, decreased hemoglobin, thrombocytopenia, and lymphocytopenia
21	10/25/2024	Orlynvah (IterumTherapeutics)	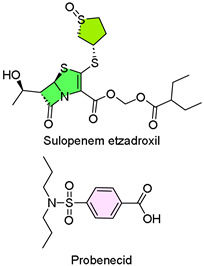	Effective treatment for Urinary tract infection (UTI)	Uncomplicated urinary tract infection (penicillin antibiotics, tubular transport inhibitor)	Diarrhea, nausea, vulvovaginal candidiasis, headache, and vomiting
22	11/15/2024	Revuforj (Syndax Pharmaceuticals)	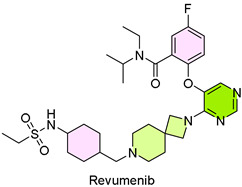	The complete response (CR) rate combined with partial hematological recovery yielded a CRh rate of 21.2%	Relapsed or refractory acute leukemia (menin inhibitor)	Bleeding, nausea, musculoskeletal pain, infection
23	11/22/2024	Rapiblyk (AOP Health)	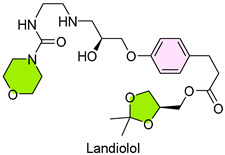	The patient’s heart rate decreases by 40% to 90%	Supraventricular tachycardia (β1-selective blocker)	Hypertension
24	11/22/2024	Attruby (Pfizer)	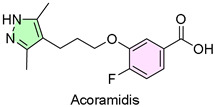	NCT03860935, NCT04622046, NCT06563895, NCT04988386, reduce cardiovascular deaths and related hospitalizations	Transthyretin stabilizer	Cardiomyopathy
25	12/13/2024	Crenessity (Neurocrine Biosciences)	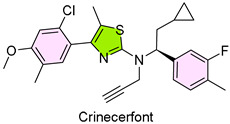	Serum androstenedione was statistically significantly lower	Congenital adrenal hyperplasia (CRF1 Antagonist)	Fatigue, headache, dizziness, joint pain, back pain, and myalgia
26	12/18/2024	Ensacove (Xcovery Holdings)	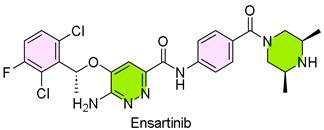	NCT02767804, the objective response rate (ORR) was 75%; 59% of patients sustained remission for 36 months or longer	Non-small cell lung cancer (ALK/MET inhibitor)	Rash, constipation, cough, pruritus, nausea, edema, fever, and fatigue
27	12/20/2024	Alyftrek (Vertex Pharmaceuticals)	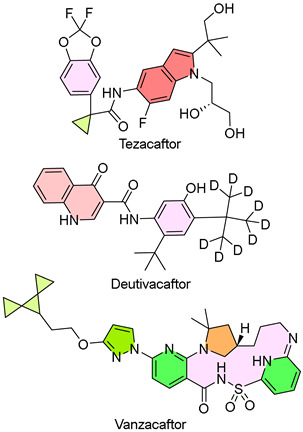	NCT05033080, NCT05076149, NCT05422222, better than Trikafta	Cystic fibrosis (CFTR, proofreader, and potentiator)	Cough, nasopharyngitis, upper respiratory tract infection, headache, oropharyngeal pain, influenza, fatigue, and rash

## Data Availability

No new data were created or analyzed in this study.
